# Consumption of whole grains and risk of type 2 diabetes: A comprehensive systematic review and dose–response meta‐analysis of prospective cohort studies

**DOI:** 10.1002/fsn3.2811

**Published:** 2022-03-10

**Authors:** Faezeh Ghanbari‐Gohari, Seyed Mohammad Mousavi, Ahmad Esmaillzadeh

**Affiliations:** ^1^ Department of Community Nutrition School of Nutritional Sciences and Dietetics Tehran University of Medical Sciences Tehran Iran; ^2^ Obesity and Eating Habits Research Center, Endocrinology and Metabolism Clinical Sciences Institute Tehran University of Medical Sciences Tehran Iran; ^3^ Food Security Research Center Department of Community Nutrition Isfahan University of Medical Sciences Isfahan Iran

**Keywords:** diabetes mellitus, meta‐analysis, systematic review, whole grain

## Abstract

This study aimed to quantitatively summarize earlier findings on the association between whole grain (WG) intake and type 2 diabetes (T2D) risk. We searched related keywords on PubMed/Medline, Scopus, and Google Scholar up to October 2021. Prospective observational studies investigating the association between WG intake and risk of T2D were included. The random‐effects model calculates the summary relative risks by contrasting categories and linear and nonlinear dose–response associations. Eleven prospective cohort studies, including 463,282 participants and 37,249 type 2 diabetes incidents, were analyzed. The pooled relative risk (RR) for the highest versus the lowest WG intake category indicated a 21% decrease in T2D risk (95% confidence interval (CI): 0.73–0.85, *I* ^2^ = 77%). Each additional 50 grams WG consumption per day was associated with a 23% reduced risk of T2D. The nonlinear association of WG and T2D revealed that 60 grams WG intake per day would give the highest benefit to prevent T2D (Pnonlinearity < 0.001). The findings were not affected by any individual study. No evidence of publication bias was documented. In conclusion, a high intake of WG was associated with a lower risk of T2D. Randomized controlled trials are needed to confirm our results.

## Graphical abstract

Type 2 diabetes, manifested by chronic hyperglycemia, has been increasing dramatically in the world. Poor diet is one of the most important lifestyle factors which is linked to the risk of diabetes. We noticed whole grains as remarkable elements of a healthy diet and reviewed earlier publications in this regard. We also quantitatively evaluated the dose–response association between total whole grain intake and incidence of type 2 diabetes in prospective cohort studies of apparently healthy adults.

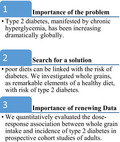

## 1 INTRODUCTION

Type 2 diabetes, manifested by chronic hyperglycemia, has been increasing dramatically in the world (Sepúlveda & Murray, 2014; Whiting et al., 2011). Approximately 463 million people have been affected by type 2 diabetes in the world up to 2019, and it is estimated that this number will reach 578 million by 2030 and 700 million by 2045 (Saeedi et al., 2019). Persistent hyperglycemia can harmfully affect microvascular and macrovascular systems and consequently result in renal and eye dysfunctions, cardiovascular disease, amputation, and other complications (Klein, 1995). In many countries, diabetes‐related health expenditures have led researchers to develop urgent diabetes prevention policies (Khan et al., 2020).

The risk of diabetes is linked to various lifestyle factors such as physical activity, obesity, alcohol, smoking, and poor diet. Among them, diet is the most important (Hu et al., 2001; Kolb & Martin, 2017). Whole grains (WG), as remarkable elements of a healthy diet (Wang et al., 2019), contain outer bran, germ, and inner endosperm of grains (Ye et al., 2012), which are rich sources of dietary fiber, antioxidants, and various micronutrients (Rebello et al., 2014). Grains are a major component of most diets, and according to the health benefits of WGs, these nutrients have attracted so much attention from health researchers (Xiao et al., 2018). Whole grains contain beneficial nutrients, including fiber, vitamins, antioxidants, and phytochemicals (Kamal‐Eldin et al., 2009; Slavin, 2003). Earlier studies suggested that whole grains can decrease blood glucose by affecting the glucose metabolism in skeletal muscles (Pereira et al., 2002). Gluco‐regulatory effects of whole grains can also be described by their high fiber content. Fiber may limit insulin secretion by slowing gastric emptying and glycemic peak (Slavin, 2003). ß‐glucans, which are barley fibers, can increase viscosity in the small intestine and delay the absorption of sugar. Consumption of these fibers reduces postprandial glycemic responses (Fardet, 2010). Greater volume and lower energy density of whole grains can also promote satiety (Koh‐Banerjee & Rimm, 2003). Compared to refined grains, whole grains are harder to digest and, therefore, they can result in lower insulin response and postprandial plasma glucose (Slavin, 2003). Documents regarding the health‐promoting effects of whole grains are conflicting. Whole grains were protectively associated with the incidence of cardiovascular diseases, some types of cancers (Gaesser, 2020), and all‐cause mortality (Aune et al., 2016; Ye et al., 2012). In contrast, some studies found no significant association between whole grain consumption and risk of stroke (Chen et al., 2016), prostate cancer (Gaesser, 2020), and breast cancer (Xiao et al., 2018). Data regarding the association of whole grain intake with type 2 diabetes is not entirely homogeneous. Several prospective studies have shown a 20%–30% reduced risk of type 2 diabetes by a greater intake of WGs or cereal fiber (Murtaugh et al., 2003), while others did not reach a significant association. Montonen et al. prospectively investigated WG and fiber consumption in relation to type 2 diabetes incidence in 4316 healthy men and women, but after ten years of follow‐up, they found a null association (Montonen et al., 2003). In a gene–diet interaction study of WG consumption and risk of type 2 diabetes, Fisher et al. did not find a statistically significant relationship between WG intake and risk of type 2 diabetes among individuals with CT + TT genotype (Fisher et al., 2009). In the two latest reviews in this regard, the investigators found a protective association between whole grain consumption and diabetes (Della Pepa et al., 2018; Wang et al., 2019). However, in one of them, the authors included only publications in the last fifteen years and combined the findings for WGs, WG foods, and diets rich in WGs (Della Pepa et al., 2018). In addition, they did not perform a dose–response analysis (Della Pepa et al., 2018). The latest dose–response analysis on this issue was done in 2017, in which 50 g/day WG intake was associated with a 25% lower risk of type 2 diabetes risk (Schwingshackl et al., 2017). However, that dose–response analysis was based on a limited number of studies due to lack of sufficient articles (Della Pepa et al., 2018). People's lifestyle has changed during recent years, which might affect the already viewed relationship between whole grain intake and risk of diabetes. As mentioned above, the previous study was done in 2017. We used the most updated data from at least five cohort populations. A big part of the data has been updated, which may affect the overall result. Several prospective cohort studies have been released after the publication of the last meta‐analysis in this regard (Ericson et al., 2018; Hu et al., 2020; Kyrø et al., 2018). Therefore, we aimed to comprehensively review earlier publications and quantitatively evaluate the dose–response association between total whole grain intake and incidence of type 2 diabetes in prospective cohort studies of apparently healthy adults.

## 2 METHODS

Guidelines of Preferred Reporting Items for Systematic Reviews and Meta‐Analyses (PRISMA) (Shamseer et al., 2015) and Meta‐analysis of Observational Studies in Epidemiology (MOOSE) (Stroup et al., 2000) were applied to report the current meta‐analysis.

### 2.1 Data sources and searches

Our search to identify relevant publications was performed in PubMed/Medline, Scopus, and Google Scholar up to October 2021. Multiple related keywords along with Medical Subject Heading (MeSH) terms (Table S1) were combined to find relevant articles. In order to prevent missing the relevant articles, we manually checked reference lists of previous accomplished reviews and included studies. In this updated comprehensive meta‐analysis, we did not limit the search in terms of languages and publication date.

### 2.2 Study selection

Included studies in the current meta‐analysis met the following specific criteria: all the studies (1) were prospective studies of cohort, case–cohort, and nested case–control design, (2) were conducted on apparently healthy adults aged >18 years, (3) had considered total whole grain consumption as the main exposure, (4) had reported type 2 diabetes incidence as the outcome, and (5) reported relative risks and 95% confidence intervals (CIs) as calculated effect sizes. To include studies in the dose–response analysis, we considered publication studies with sufficient data on the number of cases and person‐years and adjusted RRs across ≥3 categories of whole grain intake. Studies reported data on the same population were considered, and only the most updated version with a higher number of incident cases was included.

Reviews and meta‐analyses, research notes, letters, ecological studies, nonprospective observational studies, and interventional studies were excluded. We also excluded food pattern‐related articles in which whole grain intake was not considered separately.

### 2.3 Data extraction and quality assessment

To extract the required data for analysis, included eligible studies were fully reviewed by two independent authors (FG and SMM). Any discrepancies between the two investigators were resolved by consultation with the corresponding author (AE). The main data we needed was first author's name, year of publication, study location, name of the cohort, mean/median years of follow‐up, general demographic features (such as age and gender), number of participants and incident diabetes cases, method of dietary assessment, quantity of whole grain intake, and reported risk estimates (in the form of RR or HR) along with 95% CIs for each category of whole grain intake in the most adjusted model. In studies with multiple effect sizes, we pooled risk estimates using the fixed‐effect model, and the overall effect size was used for further analyses.

The primary studies' risk of bias in the current meta‐analysis was assessed using the Newcastle–Ottawa Scale (NOS) by two independent authors (SMM and FG) (Lo et al., 2014). The NOS consists of three main parts to appraise the selection of participants (4 items), the ability to compare results (2 items), and assessment of further outcomes (3 items). Overall NOS scoring ranges between 0 and 9, and studies with scores ≥7 are recognized as low risk of bias. Scores between 3 and 6 are almost acceptable, but studies that gain ≤3 scores indicating a high risk of bias.

### 2.4 Statistical analysis

We considered RRs and 95% CIs as the effect size of all included studies. Reported risks in HRs and ORs were also calculated as equal to RR (Symons & Moore, 2002). The summary RRs and 95% CIs of type 2 diabetes incidence for the highest versus lowest categories of whole grain consumption were calculated through the random‐effects models (DerSimonian & Kacker, 2007). By inversing variance of estimated logarithm, we were able to weight every study by its RR. Weighted RRs, by Der Simonian and Laird's method (25), were used to estimate average natural logarithms. We also used the random‐effects meta‐analysis to combine risk estimates in meta regression. Amounts of whole grain intakes were included as grams per day, and if a study had reported them as servings per day, we converted them to grams considering 50 g for whole grain foods and 16 grams for whole grain per serving (Ross et al., 2015). Median points of case numbers and follow‐up durations were used in analyses. Meta regressions were performed by sex, follow‐up duration, number of cases and adjustment for potential confounders (energy intake, family history, body mass index (BMI), and alcohol consumption). Heterogeneity of included studies was explored using Cochran's *Q* test (Higgins & Thompson, 2002) measured *I* squares with 25%, 50%, and 75% categorized as low, medium, and high heterogeneity. Egger's asymmetry test (Egger et al., 1997) were performed in order to examine publication bias. We also checked this bias by using a funnel plot. We checked each included study's influence on the overall effect size by performing sensitivity analyses, in which we excluded each study from overall analyses. Greenland and Longnecker's (Greenland & Longnecker, 1992) method was used to perform a linear dose–response test. This analysis required a natural log of RR estimates and 95% CIs across whole grain intake categories. We also needed specific data for this analysis, consisting of the number of participants and cases or reported person‐years and reported RRs from three or more quantitative variables. A Linear dose–response test was done on the basis of 50 g of whole grain food consumption, which is equal to one serving. Studies that reported a range of whole grain intake, we estimated the midpoint of whole grain consumption. We also used the nearest category's width to calculate the lowest or highest amounts of whole grain intake for open‐ended categories. Nonlinearity of included studies was also checked in our meta‐analysis. Number of cases or persons in each category was computed by dividing the overall reported numbers into number of categories. Categories of whole grain intake were modeled by cubic splines (Orsini et al., 2012). These models include three fixed percentiles (10%, 50%, and 90%) of whole grain consumption distribution in the overall data. We examined null hypothesis which acclaims that coefficient of middle spline is zero to calculate nonlinearity *p*‐value. Statistical significance was considered as two‐tailed *p* < .05. We used STATA software, version 15 (Stata Corp, College Station, TX) to conduct all the analyses.

## 3 RESULTS

### 3.1 Characteristics of included studies

**FIGURE 1:**
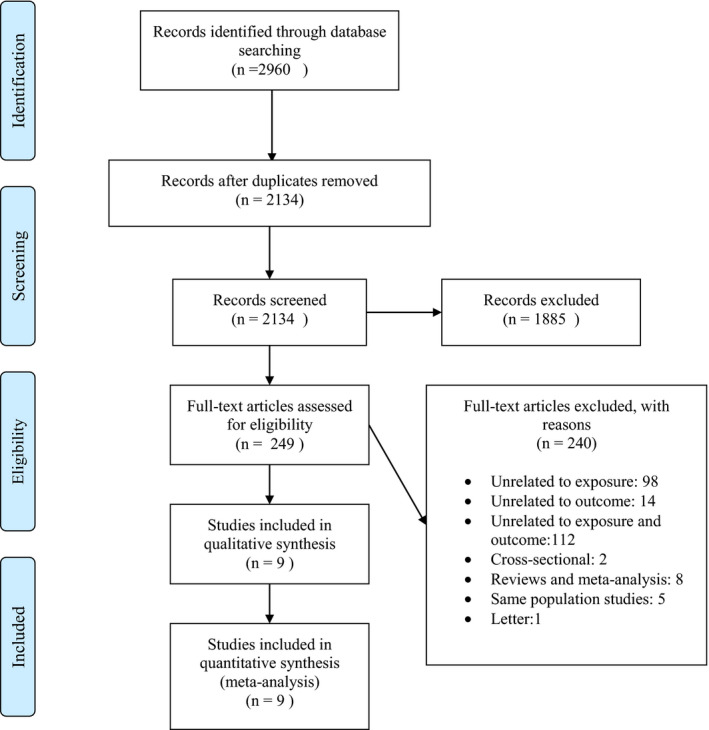
Flowchart of the number of studies identified and selected into the meta‐analysis

Based on the literature search process, shown in Figure 1, we identified 2960 publications initially. After removing duplicate articles (*n* = 826) and irrelevant publications based on screening for title and abstract (*n* = 2134), 249 citations were thoroughly reviewed, and eventually, nine articles were eligible for inclusion in the current meta‐analysis. We have explained the reasons for excluding studies in Table S2. One study had reported RRs separately for males and females (Kyrø et al., 2018) and one study for two different genotypes (Fisher et al., 2009). Therefore, these separate risk estimates were pooled using the fixed‐effect model, and the overall result of each study was used for further analyses. The study of Hu et al. had provided separate RRs for three different cohorts in the study (Hu et al., 2020). We considered each cohort of that study as a separate study, and therefore, we had 11 effect sizes from nine citations in the current meta‐analysis.

#### 3.1.1 Study characteristics

Out of 9 included publications, seven studies were of prospective cohort design (Ericson et al., 2018; Hu et al., 2020; Kyrø et al., 2018; Meyer et al., 2000; Montonen et al., 2003; Parker et al., 2013; van Dam et al., 2006), one study was as a nested case–control study (Fisher et al., 2009), and the last one was of case–cohort design (Wirström et al., 2013). Totally, 463,282 participants and 37,249 incident cases of type 2 diabetes were examined in the included studies. Most of the included studies used validated dietary assessment methods to evaluate usual WG intakes. One study did not mention the validation of their FFQ, but they used the traditional method of evaluating their questionnaire against 24‐h recalls in a smaller study population, and the results were acceptable (Kyrø et al., 2018). Most studies had used validated dietary assessment methods, either only at study baseline (5 studies) (Ericson et al., 2018; Fisher et al., 2009; Kyrø et al., 2018; Meyer et al., 2000; Montonen et al., 2003), or repeatedly (6 studies) (Hu et al., 2020; Parker et al., 2013; van Dam et al., 2006; Wirström et al., 2013) to assess WG consumption. The follow‐up duration of studies ranged from 6 to 30 years; more than half of the studies (6 studies) (Ericson et al., 2018; Hu et al., 2020; Kyrø et al., 2018; Montonen et al., 2003) followed participants for ten years or more. In total, four studies (Hu et al., 2020; Meyer et al., 2000; Parker et al., 2013; van Dam et al., 2006;) were carried out in the United States and others (5 studies) (Ericson et al., 2018; Fisher et al., 2009; Kyrø et al., 2018; Montonen et al., 2003; Wirström et al., 2013) in Europe. Most studies had controlled for alcohol consumption (*n* = 9 studies) (Ericson et al., 2018; Fisher et al., 2009; Hu et al., 2020; Kyrø et al., 2018; Meyer et al., 2000; Parker et al., 2013; van Dam et al., 2006) and body mass index (*n* = 8 studies) (Ericson et al., 2018; Fisher et al., 2009; Kyrø et al., 2018; Meyer et al., 2000; Montonen et al., 2003; Parker et al., 2013; van Dam et al., 2006; Wirström et al., 2013). Some studies adjusted for energy intake (*n* = 6 studies) (Ericson et al., 2018; Fisher et al., 2009; Meyer et al., 2000; Montonen et al., 2003; Parker et al., 2013; van Dam et al., 2006) and family history of type 2 diabetes (*n* = 6 studies) (Hu et al., 2020; Parker et al., 2013; van Dam et al., 2006; Wirström et al., 2013) as well. The characteristics of the included studies are outlined in Table 1. In addition, eight studies (Ericson et al., 2018; Hu et al., 2020; Kyrø et al., 2018; Meyer et al., 2000; Montonen et al., 2003; van Dam et al., 2006) were classified as low risk of bias (≥7 scores), and three studies (Fisher et al., 2009; Parker et al., 2013; Wirström et al., 2013) had moderate risk of bias (Table S3).

**TABLE 1:** General characteristics of the prospective cohort studies included in the meta‐analysis of whole grain intake and risk of type 2 diabetes

First Author. Year	Country	Age (range)	Sample size	Follow‐up duration (years)	Cohort name	Cases	Exposure	Dietary assessment	Comparison	HR (95% CI) (high versus low category)	Adjustments^a^	NOS
Meyer et al. (2000)	United States	55–69	F:35,988	6	IWHS	1141	WG	Baseline FFQ	Q5 versus Q1	0.79 (0.65, 0.96)	1, 4, 5, 6, 8, 17, 22, 25	7
Montonen et al. (2003)	Finland	40–69	M/F:4316	10	FMC	156	WG	Baseline FFQ	Q4 versus Q1	0.65 (0.36, 1.18)	1, 2, 4, 5, 24, 25, 32	8
Van Dam et al. (2006)	United States	21–69	F:41,186	8	BWHS	1964	WG	Repeated FFQ	C4 versus C1	0.82 (0.71–0.94)	1, 4, 5, 6, 11, 14, 17, 25, 26, 29, 30, 31	7
Fisher et al. (2009)	Germany	35–65	M/F:2318	7	EPIC‐Postdom	724	WG	Baseline FFQ	per 50g/day portion	CC genotype: 0.86 (0.75, 0.99) CT+TT genotype: 1.08 (0.96, 1.23)	1, 2, 4, 5, 6, 10, 17, 21, 23, 26, 28, 29,31	5
Wirström et al. (2013)	Sweden	35–56	F/M:4941	8–10	–	165	WG	Repeated FFQ	C3 versus C1	0.71(0.48,1.04)	1, 2, 4, 5, 9, 14, 17, 20	6
Parker et al. (2013)	United States	50–79	F:72,215	7.9	WHI	3465	WG	Repeated FFQ	C6 versus C1	0.75 (0.63, 0.89)	1, 3, 4, 5, 6, 8, 14, 15, 17, 19, 25	6
Ericson et al. (2018)	Sweden	45–74	M/F: 25,069	17	MDC	3588	WG	Baseline FFQ	T3 versus T1	0.89 (0.82–0.96)	1, 2, 4, 5, 6, 9,17, 18, 25, 27	8
Kyrø et al. (2018)	Denmark	50–65	M/F: 55,465	15	DCH	7417	WG	Baseline FFQ	Q4 versus Q1	M:0.80(0.73,0.88) F: 0.85(0.77,0.94)	4, 5, 6, 12, 17, 26	7
Hu et al. (2020)	United States	30–55	F: 69,139	30	NHS	8170	WG	Repeated FFQ	C5 versus C1	0.68(0.63, 0.73)	1, 3, 5, 6, 7, 8, 13, 14, 15, 16	7
Hu et al. (2020)	United States	25–42	F: 89,120	26	NHS II	7072	WG	Repeated FFQ	C5 versus C1	0.73 (0.68, 0.80)	1, 3, 5, 6, 7, 8, 13, 14, 15, 16.	7
Hu et al. (2020)	United States	40–75	M: 36,525	30	HPFS	3387	WG	Repeated FFQ	C5 versus C1	0.72 (0.64, 0.81)	1, 3, 5, 6, 7, 8, 13, 14, 15, 16	7

Abbreviations: BMI, body mass index; BWHS, Black Women's Health Study; C, category; CI, confidence interval; DCH, Danish Diet, Cancer, and Health cohort; EPIC‐Postdom study, European Prospective Investigation into Cancer and Nutrition; F, female; FFQ, food frequency questionnaire; FPS, Finnish prospective study; HPFS, health professional follow‐up study; HR, hazard ratio; lWHS, Iowa Women's Health Study; M, male; MDC, Malmö Diet and Cancer Study; NHS I, nurse health study I; NHS II, nurse health study II; NOS, Newcastle‐Ottawa Scale; Q, quintile or quartile; T, tertile; TEE, total energy intake; WG, whole grain.

^a^ Adjusted factors codes: 1 – Age, 2 – sex, 3 – Race/Ethnicity, 4 – BMI, 5 – Smoking status, 6 – Alcohol intake, 7 – Multivitamin use, 8 – Physical activity, 9 – Leisure time physical activity, 10 – Sports activity, 11 – Strenuous physical activity, 12 – Cambridge physical activity index, 13 – Modified alternative healthy eating index, 14 – Family history of diabetes, 15 – Postmenopausal hormone use for women, 16 – Oral contraceptive use for women, 17 – Education level, 18 – Season, 19 – Income, 20 – Blood pressure, 21 – Waist circumference, 22 – waist‐to‐hip ratio, 23 – Occupational activity, 24 – Geographic area, 25 – TEE, 26 – Red and processed meat intake, 27 – Dietary variables (according to dietary method version), 28 – butter, margarine and vegetable fat, 29 – Coffee consumption, 30 – Sugar‐sweetened soft drink consumption, 31 – Low‐fat dairy consumption, 32 – Fruit and berries, and vegetables.

### 3.2 Whole grain intake and type 2 diabetes

**FIGURE 2:**
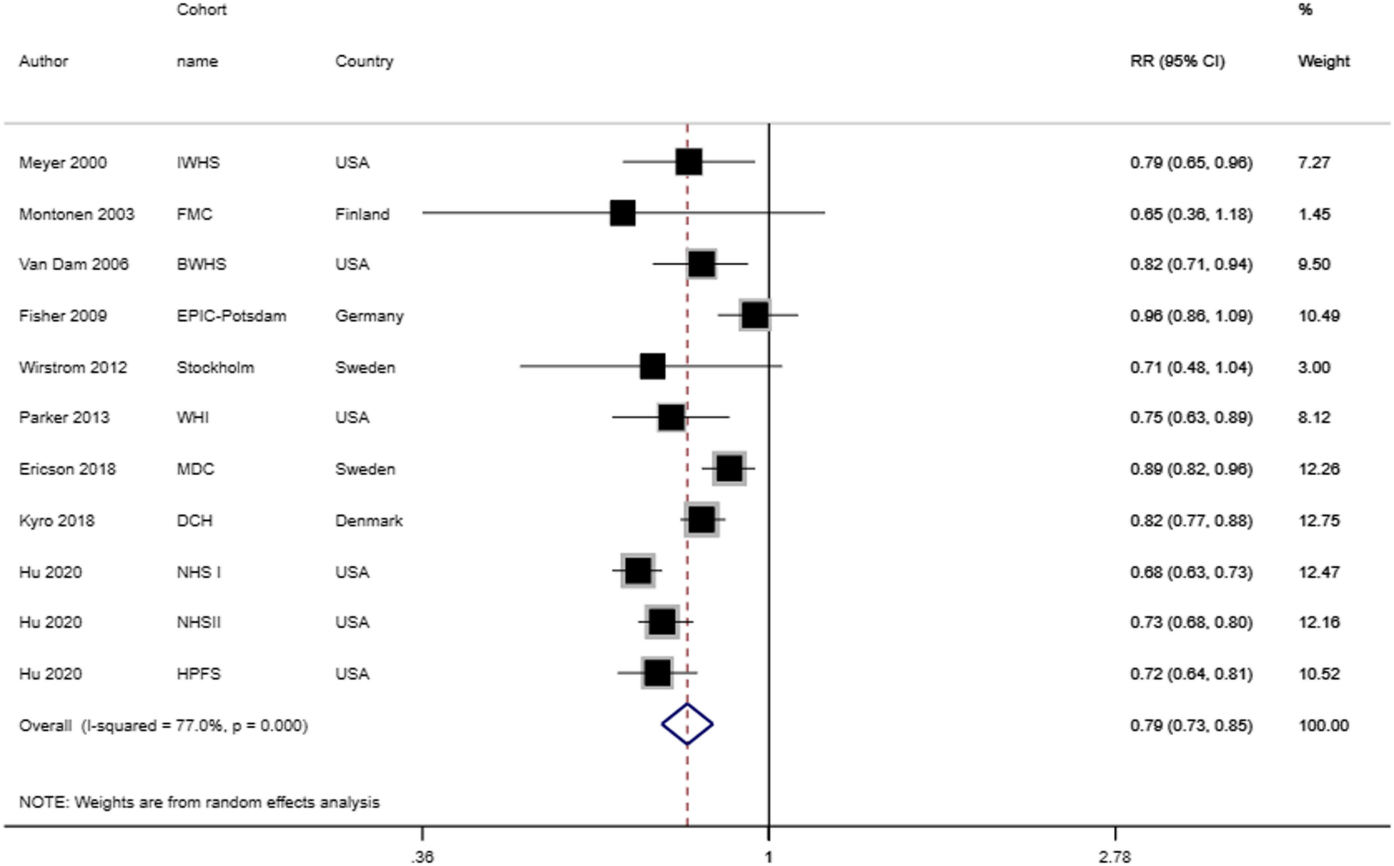
Relative risk and 95% confidence intervals (CIs) of type 2 diabetes for the highest compared to the lowest category of whole grain consumption. The black square and horizontal line represent the study‐specific HR and 95% CI, respectively; the area of the black square is proportional to the specific‐study weight to the overall meta‐analysis. The center of the open diamond presents the pooled HR and its width represents the pooled 95% CI. Weights are from random‐effects analysis

Eleven prospective cohort studies (9 publications), including 436,282 participants and 37,249 cases of type 2 diabetes, were included in this meta‐analysis. The pooled RR from the random‐effects model for comparing the highest versus lowest category of whole grain intake was 0.79 (95% CI 0.73–0.85), suggesting a 21% reduced risk of type 2 diabetes with the greatest intake of whole grains (Figure 2). There was a high between‐study heterogeneity (*I* ^2^=77% and *P*_heterogeneity_ < 0.001). We explored for the sources of this heterogeneity in subgroups with meta‐regression. We found that follow‐up duration, number of cases, location of the study, method of WG assessment (Single measurement of diet at study baseline versus repeated measurement of diet by FFQ), and adjustment for energy intake, alcohol intake, family history of type 2 diabetes, and BMI were potential sources of between‐study heterogeneity. Furthermore, in the sensitivity analysis, we found that the exclusion of individual studies one by one did not affect the overall findings, indicating our findings' robustness (Table S4, Figure S1).

Based on subgroup analysis with meta‐regression (Table 2), the overall results were regardless of location, gender, case number, follow‐up duration, method of WG assessment, and adjustment for some variables such as energy intake, alcohol consumption, BMI, and family history of type 2 diabetes; suggesting that whole grains consumption had an inverse association with risk of type 2 diabetes in all subgroups.

**TABLE 2:** Whole grain intake and the risk of type 2 diabetes (highest compared with the lowest category meta‐analysis)

Comparison	Highest versus lowest category			Dose–response (per 50 g/d)		
	No^a^	RR (95%CI)	*I* ^2^ (*p* value)	No^b^	RR (95%CI)	*I* ^2^ (*p* value)
Sex						
Men	1	0.72 (0.64, 0.81)	0	1	0.67 (0.59, 0.76)	0
Women	5	0.74 (0.66, 0.81)	41.0% (.001)	5	0.72 (0.59, 0.89)	97% (<.001)
Both	5	0.87 (0.77, 0.97)	48.6% (.03)	4	0.89 (0.80, 0.97)	72.1% (.01)
Region						
USA	6	0.73 (0.68, 0.79)	26.3% (<.001)	6	0.71 (0.60, 0.86)	96.4% (<.001)
Europe	5	0.87 (0.77, 0.97)	48.6% (.03)	4	0.89 (0.81, 0.97)	72.1% (.01)
Number of cases						
<3000	5	0.84 (0.71, 0.99)	36% (.05)	4	0.86 (0.75, 0.98)	82.8% (.001)
>3000	6	0.76 (0.68, 0.85)	83.5% (.002)	6	0.73 (0.61, 0.86)	96.2% (<.001)
Follow‐up duration						
<10 years	5	0.83 (0.71, 0.97)	48% (.03)	5	0.88 (0.80, 0.96)	79% (.001)
>10 years	6	0.76 (0.67, 0.86)	83.6% (.003)	5	0.77 (0.69, 0.87)	96% (<.001)
Assessment method adjustments						
Baseline FFQ	5	0.86% (0.78, 0.96)	47.2% (.02)	4	0.90 (0.84, 0.96)	69.1% (.02)
Repeated FFQ	6	0.72% (0.67, 0.78)	15.8% (<.001)	6	0.68 (0.55, 0.85)	96% (<.001)
Energy intake						
Yes	6	0.85 (0.76, 0.95)	40.1% (.01)	5	0.90 (0.84, 0.96)	77.1% (.002)
No	5	0.74 (0.66, 0.82)	72.3% (.002)	5	0.65 (0.53, 0.80)	92.7% (<.001)
BMI						
Yes	8	0.84 (0.78, 0.91)	35.6% (.001)	7	0.88 (0.82, 0.93)	76.2% (<.001)
No	3	0.70 (0.63, 0.79)	0% (.006)	3	0.59 (0.49, 0.71)	86% (.001)
Family history of T2D						
Yes	6	0.72 (0.67, 0.78)	15.8% (<.001)	6	0.68 (0.55, 0.85)	96% (<.001)
No	5	0.86 (0.78, 0.96)	47.2% (.02)	4	0.90 (0.85, 0.96)	69.1% (.02)
Alcohol consumption						
Yes	9	0.79 (0.72, 0.86)	81.3% (<.001)	9	0.78 (0.69, 0.89)	95% (<.001)
No	2	0.69 (0.50, 0.96)	0% (.80)	1	0.73 (0.52, 1.02)	–

Abbreviations: BMI, Body Mass Index; FFQ, Food Frequency Questionnaire; No, number; USA, United States.

^a^ Number of included studies for highest versus lowest analysis.

^b^ Number of included studies for linear dose–response analysis.

### 3.3 Dose–response meta‐analysis

**FIGURE 3:**
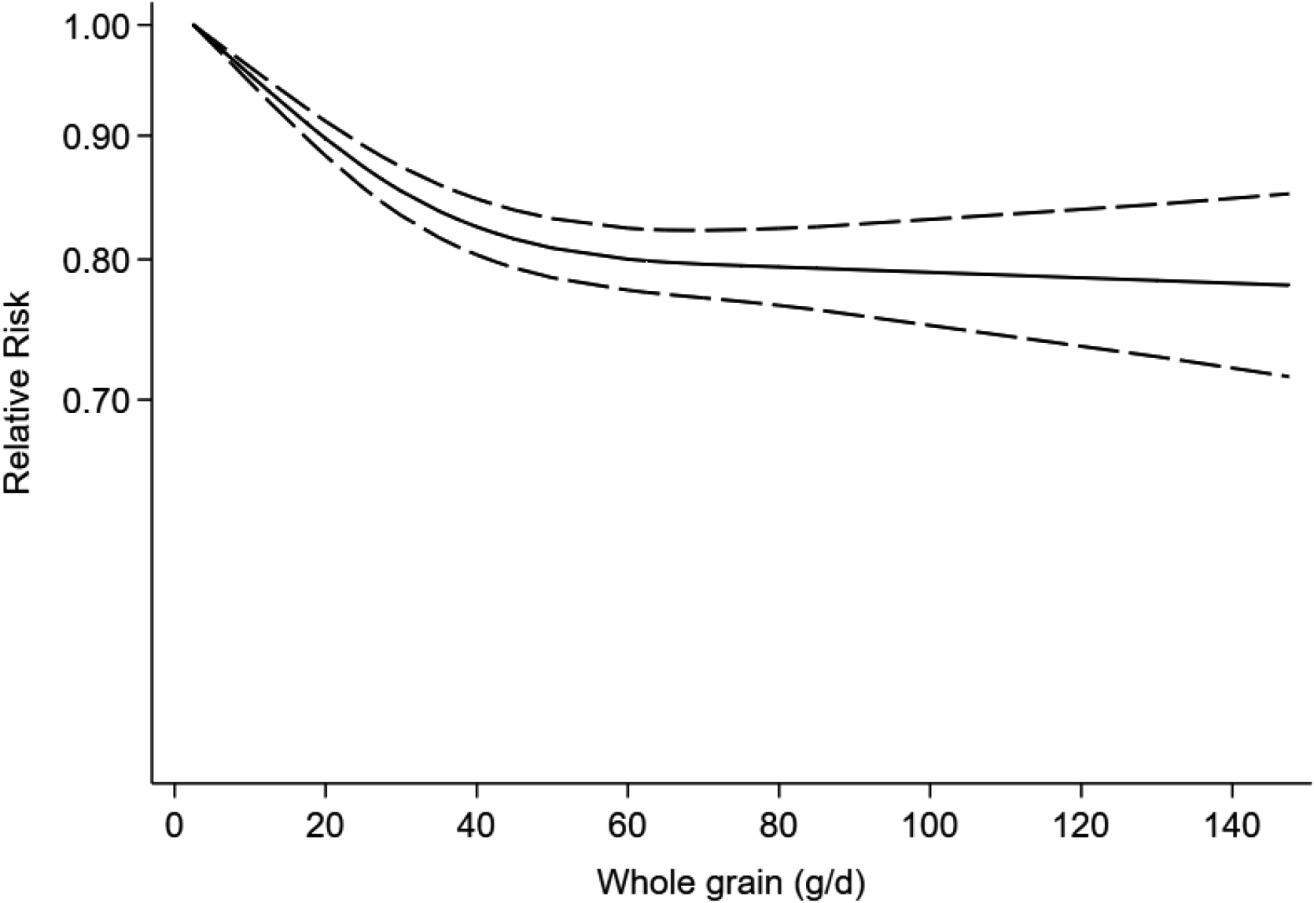
Dose–response analysis of risk of type 2 diabetes and whole grain consumption. The solid line and the long‐dashed line represent the estimated HR and its 95%CI; the solid line represents the linear relation

Nine studies from eight publications (Ericson et al., 2018; Hu et al., 2020; Kyrø et al., 2018; Meyer et al., 2000; Parker et al., 2013; van Dam et al., 2006; Wirström et al., 2013) were included in our nonlinear dose–response meta‐analysis. Based on the nonlinear dose–response analysis, whole grain consumption up to 60 g/day was remarkably associated with a lower type 2 diabetes risk, but higher amounts had slight substantial benefits (*P*_nonlinearity_ < 0.001, Figure 3, Table S5). In addition, effect sizes of ten studies from nine publications (Ericson et al., 2018; Fisher et al., 2009; Hu et al., 2020; Kyrø et al., 2018; Meyer et al., 2000; Parker et al., 2013; van Dam et al., 2006; Wirström et al., 2013) were included in linear dose–response analysis. We conducted a linear dose–response meta‐analysis by comparing risk estimates of each study per 50 g/day increments of WG. Combining effect size based on the random‐effects model showed that a 50 g/day increment in WG consumption was associated with a 23% lower risk of type 2 diabetes (RR: 0.77, 95% CI: 0.69–0.87), with a high level of heterogeneity between studies (*I* ^2^: 94.3, *P*_heterogenity_ < 0.001) (Figure S2) Due to the extremely high amounts of WG in the analysis performed by Montonen et al. (Montonen et al., 2003) and their effect on the final result, we excluded this article from both linear and nonlinear meta‐analysis to gain a more real relationship.

### 3.4 Publication bias

Assessment of publication bias by visual inspection of the funnel plot indicated no evidence of asymmetry in the association between whole grains intake and risk of type 2 diabetes (Figure S3). Also, Egger's regression test confirmed this observation (*p* = .93).

## 4 DISCUSSION

Principle findings of the current meta‐analysis of prospective cohort studies suggested that higher whole grains consumption was associated with a 21% reduction in type 2 diabetes incidence. Evidence from the dose–response analysis showed that each 50 g/day increments in WG intake might reduce the risk of type 2 diabetes by 23%. Based on the nonlinear dose–response meta‐analysis, 60 grams WG consumption was found as the optimal amount for type 2 diabetes prevention; however, higher amounts did not offer additional benefits.

Our meta‐analysis has several strengths and advantages over previous studies. We conducted a comprehensive updated meta‐analysis of WG intake in relation to type 2 diabetes incidence. Compared to prior studies, we included more relevant studies with a higher number of cases and longer follow‐up duration. More than half of included studies lasted ten years or more. Instead of WG products or specific subtypes, we tried to analyze total WG intake. We also performed both linear and nonlinear dose–response meta‐analysis on included data. The future design of the included studies has decreased the risk of recall and selection bias. According to the Newcastle–Ottawa scale (NOS), all the included papers were ranked as medium or high. However, the final result of our meta‐analysis might be affected by some limitations. First of all, published studies have reported intakes of dry weight WG (Hu et al., 2020; Kyrø et al., 2018; Wirström et al., 2013) or WG foods (Ericson et al., 2018; Fisher et al., 2009; Meyer et al., 2000; Montonen et al., 2003; Parker et al., 2013; van Dam et al., 2006) (Table S6). Although all WG intakes were converted to grams per day, some studies had not clarified WG servings in grams in the published papers (Meyer et al., 2000; Parker et al., 2013; van Dam et al., 2006). For these studies, we considered commonly used amounts of servings in the literature. Due to limited information in the published studies, we did not examine the association of subtypes of WGs (dark bread, brown rice, etc.) with diabetes. Due to the inclusion of populations with various characteristics, we found a high level of heterogeneity. It must also be kept in mind that higher WG consumption is related to a healthier lifestyle. All confounding variables, such as physical activity, might not have been adjusted in the included studies. Therefore, our findings might have been affected by some potential unmeasured or residual confounders. Although most the studies used validated FFQs to assess usual WG intakes, we cannot deny the possibility of error in participants' self‐reports. Some included studies have measured WG's intakes at study baseline only, which might be changed during follow‐up. Since a small proportion of participants consumed more than 60 grams of whole grains per day, the plateau observed in the dose–response curve could not be considered a definitive finding. Due to the limited data available in this study, it was impossible to investigate further the association between high whole grain intakes and the risk of diabetes. In some included studies, the diagnosis of type 2 diabetes was based on a questionnaire, which can be less accurate.

A meta‐analysis of randomized clinical trials revealed that WG intake might beneficially affect systematic inflammation (Hajihashemi & Haghighatdoost, 2019); however, comprehensive analysis in this regard revealed no significant effect of WG intake on inflammatory biomarkers (Rahmani et al., 2020; Sang et al., 2020). The impact of WG consumption on glycemic control has also been explored in a meta‐analysis of RCTs (Marventano et al., 2017), in which the authors reported effective controlling of postprandial blood glucose and insulin hemostasis by WG intake (Marventano et al., 2017). Given the role of obesity in most chronic diseases, the association of WG intake with bodyweight was also investigated (Maki et al., 2019). Findings from observational studies revealed lower body weight among people with a high WG intake (Maki et al., 2019); however, clinical trial studies demonstrated no beneficial effect of WG on body weight and other obesity measures (Maki et al., 2019). In the current study, we updated previous meta‐analyses about WG intake and risk of type 2 diabetes risk. We found a significant inverse association between WG consumption and type 2 diabetes incidence (RR: 0.79; 95% CI: 0.73–0.85). Our findings were the same comparing to earlier meta‐analyses. Wang et al. conducted a meta‐analysis on eight studies (Wang et al., 2019) and found a 32% reduced risk of type 2 diabetes incidence with high WG intake (RR:0.68, 95% CI: 0.64–0.73) (Wang et al., 2019). The difference between Wang et al. analysis and ours is that we included studies on total WG consumption, while they included various individual WG products in their research. In a systematic review of observational and interventional studies on WG intake and type 2 diabetes in 2018 (Della Pepa et al., 2018), the investigators concluded that all observational studies showed the inverse relationship between WG consumption and type 2 diabetes. In contrast, they failed to find such association for interventional studies due to lack of related articles (19). In a meta‐analysis in 2017 by Schwingshackl et al. (2017), WG intake was inversely associated with type 2 diabetes (RR: 0.77; 95% CI 0.71–0.84; Schwingshackl et al., 2017). They found a 25% reduction in type 2 diabetes incidence by each additional 50‐gram intake of WG per day (Schwingshackl et al., 2017). Although a considerable part of our data has been updated, our overall result was in agreement with previous studies. The difference between our study and that of Schwingshackl et al. was that we updated the data by including two recent prospective studies about this subject (Ericson et al., 2018; Kyrø et al., 2018). We also used updated data of the United States' three big cohorts (NHS I, NHS II, and HPFS) (Hu et al., 2020) to get more accurate data. Another meta‐analysis on WG intake, refined grains, and their subtypes with the risk of type 2 diabetes was published in 2013, in which an inverse association of WG with the incidence of type 2 diabetes was documented (RR:0.68, 95% CI 0.58–0.81) (Aune et al., 2013). They analyzed different subtypes of WG and refined grain with type 2 diabetes, but due to insufficient studies, the investigators claimed that the findings were not so strong to rely on. Due to the lack of enough data on intakes of whole grain subtypes in different cohort populations, we tried to analyze overall whole grain intake in various studies. To reach a more stable relationship, we also tried to consider the amount of whole grain in foods instead of whole grain products.

Potential mechanisms of the association between WG intake and reduced risk of type 2 diabetes might be related to their nutrient content. WGs include fibers, phytochemicals, vitamins, minerals, lignans, or phytic acid (Fardet, 2010). Dietary fiber can affect body weight (Slavin, 2005), through which it can influence the risk of type 2 diabetes. Prior investigations have shown reduced weight gain in people who consume more WGs (Liu et al., 2003). In addition, insoluble fibers have a rough structure that increases chewing food, resulting in satiety (Wanders et al., 2011). Dietary fiber can also prevent type 2 diabetes by improving insulin sensitivity in the body (Weickert & Pfeiffer, 2018). Viscous fibers can also positively affect blood glucose by stimulating satiety signals and related hormones (Chutkan et al., 2012). Soluble fibers can control postprandial blood glucose inducing a delay in gastric emptying, increasing the transit time and absorption of glucose (Lattimer & Haub, 2010). Besides fiber content, WGs can reduce type 2 diabetes risk by lowering inflammatory markers such as C‐reactive protein (Qi et al., 2006). Higher levels of some liver enzymes, such as aspartate aminotransferase, can lead the body to be more susceptible to type 2 diabetes, but these markers can be controlled in normal ranges by WG consumption (Choi et al., 2020; Kim et al., 2009). WG and cereal fibers are associated with a greater adiponectin concentration, which is known to control weight and increase insulin sensitivity (Li et al., 2009; Qi et al., 2006). Moreover, insoluble fibers are digested by the bacterial gut population and produce short‐chain fatty acids (SCFAs) (Hernández et al., 2019), which can, in turn, mediate the secretion of gut hormones and beneficially affect glucose and lipid metabolism (Bach Knudsen, 2015; Hernández et al., 2019). Findings from a previous meta‐analysis confirmed that WG consumption was associated with a lower concentration of fasting blood sugar and insulin (Nettleton et al., 2010). WGs are rich in magnesium, a mineral that seems to improve the metabolism of glucose and prevent type 2 diabetes (Kim et al., 2010; Song et al., 2006; Volpe, 2008).

In conclusion, the current meta‐analysis demonstrated that WG consumption was inversely associated with the risk of type 2 diabetes incidence. Higher WG intake was associated with a 23% lower occurrence of type 2 diabetes. We also found that the optimal intake of whole grains was about 50–60 grams per day. Given the basis of this review on observational studies, further studies, in particular long‐term randomized clinical trials, are necessary to reach a causal relationship.

## CONFLICT OF INTEREST

The authors declare that they do not have any conflict of interest.

## ETHICAL APPROVAL

This study does not involve any human or animal testing.

## Supporting information

Appendix S1

## Data Availability

The data that support the findings of this study are available in the Appendix S1 of this article.
