# Supplementary material for: Consumption of whole grains and risk of type 2 diabetes: A comprehensive systematic review and dose–response meta‐analysis of prospective cohort studies
Source: Food Sci Nutr. 2022 Mar 10;10(6):1950–60. doi: 10.1002/fsn3.2811 (PMC9179146; doi:10.1002/fsn3.2811)
Supplement: Supplementary file 1 — Appendix S1 [file FSN3-10-1950-s001.docx]

**Consumption of whole grains and risk of type 2 diabetes: a comprehensive systematic review and dose-response meta-analysis of prospective cohort studies**

**Faezeh Ghanbari-Gohari^1^, Seyed Mohammad Mousavi^1^, Ahmad Esmaillzadeh^1,2,3^***

*^1^ Department of Community Nutrition, School of Nutritional Sciences and Dietetics, Tehran University of Medical Sciences, Tehran, Iran*

*^2^ Obesity and Eating Habits Research Center, Endocrinology and Metabolism Molecular Cellular Sciences Institute, Tehran University of Medical Sciences, Tehran, Iran*

*^3^ Food Security Research Center, Department of Community Nutrition, Isfahan University of Medical Sciences, Isfahan, Iran*

**Correspondence to:**

Ahmad Esmaillzadeh, Ph.D.

Department of Community Nutrition,

School of Nutritional Sciences and Dietetics,

Tehran University of Medical Sciences, Tehran, Iran

P.O. Box 14155-6117

Tel: +98-21-88955805

Fax: +98-21-88984861

Email: [a-esmaillzadeh@tums.ac.ir](mailto:a-esmaillzadeh@tums.ac.ir)

**ORCID**: <https://orcid.org/0000-0002-5547-7549>

**Running Title**: Whole grains and diabetes

**KEYWORDS:** Diabetes, Diabetes mellitus, Dose-response, Meta-analysis, Systematic review, Diabetes type 2, Whole grain

Supplementary data including 6 supplemental Tables, and 3 supplemental Figures

**Supplemental Tables**

| **Supplemental Table 1.** Search strategy to find the relevant articles for inclusion in this meta-analysis |
| --- |
| **Pubmed**  **"Whole Grain"[MeSH Terms] OR "Triticale"[MeSH Terms] OR "Millets"[MeSH Terms] OR "Dietary fiber"[MeSH Terms] OR "Edible grain"[MeSH Terms] OR "Triticum"[MeSH Terms] OR "Whole Grain"[Title/Abstract] OR "Triticale"[Title/Abstract] OR "Millets"[Title/Abstract] OR "Dietary fiber"[Title/Abstract] OR "Edible grain"[Title/Abstract] OR "Triticum"[Title/Abstract] OR "Wholegrain*"[Title/Abstract] OR "Whole grain*"[Title/Abstract] OR "Whole-grain"[Title/Abstract] OR "Whole-grains"[Title/Abstract] OR "Whole grain rice"[Title/Abstract] OR "Whole-grain rice"[Title/Abstract] OR "Whole grain cereal"[Title/Abstract] OR "whole grain cereals"[Title/Abstract] OR "Cereal grain"[Title/Abstract] OR "Cereal grains"[Title/Abstract] OR "Grain"[Title/Abstract] OR "Cereal"[Title/Abstract] OR "Cereals"[Title/Abstract] OR Oat[Title/Abstract] OR Oats[Title/Abstract] OR "Cultivated oat"[Title/Abstract] OR "Cultivated oats"[Title/Abstract] OR "Wheat bran"[Title/Abstract] OR "Wheat brans"[Title/Abstract] OR Bran[Title/Abstract] OR "Brown rice"[Title/Abstract]**  **AND**  **"Diabetes Mellitus"[MeSH Terms] OR "Diabetes Mellitus, Type 2"[MeSH Terms] OR "Insulin resistance"[MeSH Terms] OR "Hyperglycemia"[MeSH Terms] OR "Diabetes Mellitus"[Title/Abstract] OR "Diabetes Mellitus, Type 2"[Title/Abstract] OR "Insulin resistance"[Title/Abstract] OR Hyperglycemia[Title/Abstract] OR Dibetes[Title/Abstract] OR Diabetic[Title/Abstract] OR "Diabetes type 2"[Title/Abstract] OR "Type II diabetes"[Title/Abstract] OR "Type 2 diabets"[Title/Abstract] OR "Type 2 diabetes mellitus"[Title/Abstract] OR "Non-insulin dependent diabetes"[Title/Abstract] OR NIDDM[Title/Abstract] OR "Diabetes mellitus non-insulin dependent"[Title/Abstract] OR "Diabetes non-insulin dependent"[Title/Abstract] OR "Non-insulin-dependent diabetes mellitus"[Title/Abstract] OR "Impaired glucose tolerance"[Title/Abstract] OR "Impaired fasting glucose"[Title/Abstract] OR "Impaired plasma glucose"[Title/Abstract] OR "Glucose intolerance"[Title/Abstract] OR "Impaired[Title/Abstract] AND fasting blood sugar"[Title/Abstract] OR IGT[Title/Abstract] OR IFG[Title/Abstract] OR Hyperglycemias[Title/Abstract] OR "Postprandial hyperglycemias"[Title/Abstract] OR "Postprandial hyperglycemia"[Title/Abstract] OR "Glycemic control"[Title/Abstract] OR "Latent diabetes"[Title/Abstract] OR "Diabetes risk"[Title/Abstract] OR "Stable diabetes mellitus"[Title/Abstract] OR "Maturity-onset diabetes mellitus"[Title/Abstract] OR "Maturity onset diabetes mellitus"[Title/Abstract] OR MODY[Title/Abstract] OR "Slow-onset diabetes mellitus "[Title/Abstract] OR "Noninsulin-dependent diabetes mellitus"[Title/Abstract] OR "Noninsulin dependent diabetes mellitus"[Title/Abstract] OR "Maturity-onset diabetes"[Title/Abstract] OR "Maturity onset diabetes"[Title/Abstract] OR "Adult-onset diabetes mellitus"[Title/Abstract]**  **AND**  **"Cohort Studies"[MeSH Terms] OR "Observation"[MeSH Terms] OR "Prospective Studies"[MeSH Terms] OR "Longitudinal Studies"[MeSH Terms] OR "Observational Study"[MeSH Terms] OR "Follow-Up Studies"[MeSH Terms] OR "Case-Control Studies"[MeSH Terms] OR "Cohort Studies"[Title/Abstract] OR Observation[Title/Abstract] OR "Prospective Studies"[Title/Abstract] OR "Longitudinal Studies"[Title/Abstract] OR "Observational Study"[Title/Abstract] OR "Follow-Up Studies"[Title/Abstract] OR "Case-Control Studies"[Title/Abstract] OR Cohorts[Title/Abstract] OR Cohort[Title/Abstract] OR "Cohort Studies"[Title/Abstract] OR "Concurrent Study"[Title/Abstract] OR "Incidence Studies"[Title/Abstract] OR Observation[Title/Abstract] OR "Prospective Studies"[Title/Abstract] OR "Prospectively"[Title/Abstract] OR Prospective[Title/Abstract] OR Longitudinal[Title/Abstract] OR Observational[Title/Abstract] OR "Follow-Up "[Title/Abstract] OR Nested[Title/Abstract] OR "Relative risk"[Title/Abstract] OR "Hazard ratio"[Title/Abstract] OR RR[Title/Abstract] OR HR[Title/Abstract]** |
| **scopus**  **(** **(** TITLE-ABS-KEY *(*"Whole-grain"*)*  OR  TITLE-ABS-KEY ( "Whole-grains" )  OR  TITLE-ABS-KEY ( "Whole grain rice" )  OR  TITLE-ABS-KEY ( "Whole-grain rice" )  OR  TITLE-ABS-KEY ( "Whole grain cereal " )  OR  TITLE-ABS-KEY ( "whole grain cereals" )  OR  TITLE-ABS-KEY ( "Cereal grain" )  OR  TITLE-ABS-KEY ( "Cereal grains" )  OR  TITLE-ABS-KEY ( grain )  OR  TITLE-ABS-KEY ( cereal )  OR  TITLE-ABS-KEY ( cereals )  OR  TITLE-ABS-KEY ( oat )  OR  TITLE-ABS-KEY ( oats )  OR  TITLE-ABS-KEY ( "Cultivated oat" )  OR  TITLE-ABS-KEY ( "Cultivated oats" )  OR  TITLE-ABS-KEY ( "Wheat bran" )  OR  TITLE-ABS-KEY ( "Wheat brans" )  OR  TITLE-ABS-KEY ( bran )  OR  TITLE-ABS-KEY ( "Brown rice" ) OR TITLE-ABS-KEY (“Whole Grain”) OR TITLE-ABS-KEY (Triticale) OR TITLE-ABS-KEY (Millets) OR TITLE-ABS-KEY (Dietary fiber) OR TITLE-ABS-KEY (“Edible grain”) OR TITLE-ABS-KEY (Triticum) ) )  AND  **(** **(** TITLE-ABS-KEY ( "Dibetes" )  OR  TITLE-ABS-KEY ( "Diabetic" )  OR  TITLE-ABS-KEY ( "Diabetes type 2" )  OR  TITLE-ABS-KEY ( "Type II diabetes" )  OR  TITLE-ABS-KEY ( "Type 2 diabets" )  OR  TITLE-ABS-KEY ( "Type 2 diabetes mellitus" )  OR  TITLE-ABS-KEY ( "Non-insulin dependent diabetes" )  OR  TITLE-ABS-KEY ( niddm )  OR  TITLE-ABS-KEY ( "Diabetes mellitus non-insulin dependent" )  OR  TITLE-ABS-KEY ( "Diabetes non-insulin dependent" )  OR  TITLE-ABS-KEY ( "Non-insulin-dependent diabetes mellitus" )  OR  TITLE-ABS-KEY ( "Impaired glucose tolerance" )  OR  TITLE-ABS-KEY ( "Impaired fasting glucose" )  OR  TITLE-ABS-KEY ( "Impaired plasma glucose" )  OR  TITLE-ABS-KEY ( "Glucose intolerance" )  OR  TITLE-ABS-KEY ( "Impaired and fasting blood sugar" )  OR  TITLE-ABS-KEY ( igt )  OR  TITLE-ABS-KEY ( ifg )  OR  TITLE-ABS-KEY ( hyperglycemias )  OR  TITLE-ABS-KEY ( "Postprandial hyperglycemias" )  OR  TITLE-ABS-KEY ( "Postprandial hyperglycemia" )  OR  TITLE-ABS-KEY ( "Glycemic control" )  OR  TITLE-ABS-KEY ( "Latent diabetes" )  OR  TITLE-ABS-KEY ( "Diabetes risk" )  OR  TITLE-ABS-KEY ( "Stable diabetes mellitus" )  OR  TITLE-ABS-KEY ( "Maturity-onset diabetes mellitus" )  OR  TITLE-ABS-KEY ( "Maturity onset diabetes mellitus" )  OR  TITLE-ABS-KEY ( mody )  OR  TITLE-ABS-KEY ( "Slow-onset diabetes mellitus " )  OR  TITLE-ABS-KEY ( "Noninsulin-dependent diabetes mellitus" )  OR  TITLE-ABS-KEY ( "Noninsulin dependent diabetes mellitus" )  OR  TITLE-ABS-KEY ( "Maturity-onset diabetes"*)* OR  TITLE-ABS-KEY ( "Maturity onset diabetes" )  OR  TITLE-ABS-KEY ( "Adult-onset diabetes mellitus" ) OR TITLE-ABS-KEY (“Diabetes Mellitus”) OR TITLE-ABS-KEY (“Diabetes Mellitus, Type 2”) OR TITLE-ABS-KEY (“Insulin resistance”) OR TITLE-ABS-KEY (Hyperglycemia) ) )  AND  **(** **(** TITLE-ABS-KEY ( cohorts )  OR  TITLE-ABS-KEY ( cohort )  OR  TITLE-ABS-KEY ( "Cohort Studies" )  OR  TITLE-ABS-KEY ( "Concurrent Study" )  OR  TITLE-ABS-KEY ( "Incidence Studies" )  OR  TITLE-ABS-KEY ( observation )  OR  TITLE-ABS-KEY ( "Prospective Studies" )  OR  TITLE-ABS-KEY ( prospectively )  OR  TITLE-ABS-KEY ( prospective )  OR  TITLE-ABS-KEY ( longitudinal )  OR  TITLE-ABS-KEY ( observational )  OR  TITLE-ABS-KEY ( "Follow-Up" )  OR  TITLE-ABS-KEY ( nested )  OR  TITLE-ABS-KEY ( "Relative risk" )  OR  TITLE-ABS-KEY ( "Hazard ratio**"** )  OR  TITLE-ABS-KEY ( rr )  OR  TITLE-ABS-KEY ( hr ) OR TITLE-ABS-KEY  (“Cohort Studies”) OR TITLE-ABS- KEY (Observation) OR TITLE-ABS-KEY (“Prospective Studies”) OR TITLE-ABS-KEY  (“Longitudinal Studies”) OR TITLE-ABS-KEY (“Observational Study”) OR TITLE-ABS-KEY  (“Follow-Up Studies”) OR TITLE-ABS-KEY  (“Case-Control Studies”) ) ). |

| **Supplemental Table 2**: Reason for exclusion of retrieved articles |  |
| --- | --- |
| References | reason for exclusion |
| 1. Pereira, M. A., et al. (1998). "The Association of Whole Grain Intake and Fasting Insulin in a Biracial Cohort of Young Adults: The CARDIA Study." CVD Prev **1**(3): 231-242. | unrelated outcome |
| 1. The effect of oat bran consumption on gestational diabetes: a randomized controlled clinical trial | unrelated outcome |
| 1. Steffen, L. M., et al. (2003). "Whole grain intake is associated with lower body mass and greater insulin sensitivity among adolescents." Am J Epidemiol **158**(3): 243-250. | unrelated outcome |
| 1. Whole- and Refined-Grain Consumption and Longitudinal Changes in Cardiometabolic Risk Factors in the Framingham Offspring Cohort | unrelated outcome |
| 1. Whole-grain processing and glycemic control in type 2 diabetes: A randomized crossover trial | unrelated outcome |
| 1. Whole Grain Intake and Impaired Fasting Glucose in Adolescents, National Health and Nutrition Examination Survey, 2005-2014 | unrelated outcome |
| 1. Grain and dietary fiber intake and bladder cancer risk: a pooled analysis of prospective cohort studies | unrelated outcome |
| 1. Higher Alkylresorcinol Concentrations, a Consequence of Whole-Grain Intake, are Inversely Associated with Gestational Diabetes Mellitus in Iceland | unrelated outcome |
| 1. McKeown, N. M., et al. (2004). "Carbohydrate nutrition, insulin resistance, and the prevalence of the metabolic syndrome in the Framingham Offspring Cohort." Diabetes Care **27**(2): 538-546. | unrelated outcome |
| 1. Zhang, C., et al. (2006). "Dietary fiber intake, dietary glycemic load, and the risk for gestational diabetes mellitus." Diabetes Care **29**(10): 2223-2230. | unrelated outcome |
| 1. Kristensen, M., et al. (2012). "Whole grain compared with refined wheat decreases the percentage of body fat following a 12-week, energy-restricted dietary intervention in postmenopausal women." Journal of Nutrition **142**(4): 710-716. | unrelated outcome |
| 1. Mhd Omar, N. A., et al. (2020). "Long-term whole-grain rye and wheat consumption and their associations with selected biomarkers of inflammation, endothelial function, and cardiovascular disease." Eur J Clin Nutr. | unrelated outcome |
| 1. The effect of a brown-rice diets on glycemic control and metabolic parameters in prediabetes and type 2 diabetes mellitus: a meta-analysis of randomized controlled trials and controlled clinical trials | unrelated outcome |
| 1. Dietary fibre and whole grains in diabetes management: Systematic review and meta-analyses | unrelated outcome |
| 1. Hu, F. B., et al. (2001). "Diet, lifestyle, and the risk of type 2 diabetes mellitus in women." N Engl J Med **345**(11): 790-797. | unrelated exposure |
| 1. Stevens, J., et al. (2002). "Dietary fiber intake and glycemic index and incidence of diabetes in African-American and white adults: the ARIC study." Diabetes Care **25**(10): 1715-1721. | unrelated exposure |
| 1. Schulze, M. B., et al. (2003). "Processed meat intake and incidence of Type 2 diabetes in younger and middle-aged women." Diabetologia **46**(11): 1465-1473. | unrelated exposure |
| 1. Wannamethee, S. G., et al. (2003). "Alcohol drinking patterns and risk of type 2 diabetes mellitus among younger women." Arch Intern Med **163**(11): 1329-1336. | unrelated exposure |
| 1. Fung, T. T., et al. (2004). "Dietary patterns, meat intake, and the risk of type 2 diabetes in women." Arch Intern Med **164**(20): 2235-2240. | unrelated exposure |
| 1. Lopez-Ridaura, R., et al. (2004). "Magnesium intake and risk of type 2 diabetes in men and women." Diabetes Care **27**(1): 134-140. | unrelated exposure |
| 1. Hodge, A. M., et al. (2004). "Glycemic index and dietary fiber and the risk of type 2 diabetes." Diabetes Care **27**(11): 2701-2706. | unrelated exposure |
| 1. Schulze, M. B., et al. (2004). "Glycemic index, glycemic load, and dietary fiber intake and incidence of type 2 diabetes in younger and middle-aged women." Am J Clin Nutr **80**(2): 348-356. | unrelated exposure |
| 1. Song, Y., et al. (2004). "A prospective study of red meat consumption and type 2 diabetes in middle-aged and elderly women: the women's health study." Diabetes Care **27**(9): 2108-2115. | unrelated exposure |
| 1. Song, Y., et al. (2004). "Dietary Magnesium Intake in Relation to Plasma Insulin Levels and Risk of Type 2 Diabetes in Women." Diabetes Care **27**(1): 59-65. | unrelated exposure |
| 1. Schulze, M. B., et al. (2005). "Dietary pattern, inflammation, and incidence of type 2 diabetes in women." American Journal of Clinical Nutrition **82**(3): 675-684. | unrelated exposure |
| 1. Shai, I., et al. (2006). "Ethnicity, obesity, and risk of type 2 diabetes in women: a 20-year follow-up study." Diabetes Care **29**(7): 1585-1590. | unrelated exposure |
| 1. Barclay, A. W., et al. (2007). "Glycemic index, dietary fiber, and risk of type 2 diabetes in a cohort of older Australians." Diabetes Care **30**(11): 2811-2813. | unrelated exposure |
| 1. Krishnan, S., et al. (2007). "Glycemic index, glycemic load, and cereal fiber intake and risk of type 2 diabetes in US black women." Arch Intern Med **167**(21): 2304-2309. | unrelated exposure |
| 1. Sahyoun, N. R., et al. (2008). "Dietary glycemic index and glycemic load and the risk of type 2 diabetes in older adults." Am J Clin Nutr **87**(1): 126-131. | unrelated exposure |
| 1. Schulze, M. B., et al. (2007). "Fiber and magnesium intake and incidence of type 2 diabetes: a prospective study and meta-analysis." Arch Intern Med **167**(9): 956-965. | unrelated exposure |
| 1. Imamura, F., et al. (2009). "Generalizability of dietary patterns associated with incidence of type 2 diabetes mellitus." American Journal of Clinical Nutrition **90**(4): 1075-1083. | unrelated exposure |
| 1. Liese, A. D., et al. (2009). "Food intake patterns associated with incident type 2 diabetes: the Insulin Resistance Atherosclerosis Study." Diabetes Care **32**(2): 263-268. | unrelated exposure |
| 1. Wannamethee, S. G., et al. (2009). "Associations between dietary fiber and inflammation, hepatic function, and risk of type 2 diabetes in older men: potential mechanisms for the benefits of fiber on diabetes risk." Diabetes Care **32**(10): 1823-1825. | unrelated exposure |
| 1. Hopping, B. N., et al. (2010). "Dietary fiber, magnesium, and glycemic load alter risk of type 2 diabetes in a multiethnic cohort in Hawaii." J Nutr **140**(1): 68-74. | unrelated exposure |
| 1. Kochar, J., et al. (2010). "Nut consumption and risk of type II diabetes in the Physicians Health Study." Eur J Clin Nutr **64**(1): 75-79. | unrelated exposure |
| 1. Nanri, A., et al. (2010). "Rice intake and type 2 diabetes in Japanese men and women: the Japan Public Health Center-based Prospective Study." Am J Clin Nutr **92**(6): 1468-1477. | unrelated exposure |
| 1. Sluijs, I., et al. (2010). "Carbohydrate quantity and quality and risk of type 2 diabetes in the European Prospective Investigation into Cancer and Nutrition-Netherlands (EPIC-NL) study." American Journal of Clinical Nutrition **92**(4): 905-911. | unrelated exposure |
| 1. Malik, V. S., et al. (2011). "Adolescent dairy product consumption and risk of type 2 diabetes in middle-aged women." American Journal of Clinical Nutrition **94**(3): 854-861. | unrelated exposure |
| 1. Pan, A., et al. (2011). "Red meat consumption and risk of type 2 diabetes: 3 cohorts of US adults and an updated meta-analysis." Am J Clin Nutr **94**(4): 1088-1096. | unrelated exposure |
| 1. Yu, R., et al. (2011). "Relationship between dietary intake and the development of type 2 diabetes in a Chinese population: The Hong Kong Dietary Survey." Public Health Nutr **14**(7): 1133-1141. | unrelated exposure |
| 1. Hindy, G., et al. (2012). "Role of TCF7L2 risk variant and dietary fibre intake on incident type 2 diabetes." Diabetologia **55**(10): 2646-2654. | unrelated exposure |
| 1. Lajous, M., et al. (2012). "Processed and unprocessed red meat consumption and incident type 2 diabetes among French women." Diabetes Care **35**(1): 128-130. | unrelated exposure |
| 1. Malik, V. S., et al. (2012). "Dietary patterns during adolescence and risk of type 2 diabetes in middle-aged women." Diabetes Care **35**(1): 12-18. | unrelated exposure |
| 1. Kimura, Y., et al. (2020). "Dietary fiber intake and risk of type 2 diabetes in a general Japanese population: The Hisayama Study." J Diabetes Investig. | unrelated exposure |
| 1. Ericson, U., et al. (2019). "Food patterns in relation to weight change and incidence of type 2 diabetes, coronary events and stroke in the Malmö Diet and Cancer cohort." Eur J Nutr **58**(5): 1801-1814. | unrelated exposure |
| 1. Zong, G., et al. (2018). "Gluten intake and risk of type 2 diabetes in three large prospective cohort studies of US men and women." Diabetologia **61**(10): 2164-2173. | unrelated exposure |
| 1. Shan, R., et al. (2018). "Low-Carbohydrate, High-Protein, High-Fat Diets Rich in Livestock, Poultry and Their Products Predict Impending Risk of Type 2 Diabetes in Chinese Individuals that Exceed Their Calculated Caloric Requirement." Nutrients **10**(1). | unrelated exposure |
| 1. Li, S. X., et al. (2018). "Interplay between genetic predisposition, macronutrient intake and type 2 diabetes incidence: analysis within EPIC-InterAct across eight European countries." Diabetologia **61**(6): 1325-1332. | unrelated exposure |
| 1. O’Connor, L. E., et al. (2020). "Adherence to a Mediterranean-style eating pattern and risk of diabetes in a U.S. prospective cohort study." Nutrition and Diabetes **10**(1). | unrelated exposure |
| 1. Abiemo, E. E., et al. (2013). "Relationships of the Mediterranean dietary pattern with insulin resistance and diabetes incidence in the Multi-Ethnic Study of Atherosclerosis (MESA)." Br J Nutr **109**(8): 1490-1497. | unrelated exposure |
| 1. Virtanen, H. E. K., et al. (2017). "Intake of different dietary proteins and risk of type 2 diabetes in men: The Kuopio Ischaemic Heart Disease Risk Factor Study." British Journal of Nutrition **117**(6): 882-893. | unrelated exposure |
| 1. Lv, J., et al. (2017). "Adherence to a healthy lifestyle and the risk of type 2 diabetes in Chinese adults." Int J Epidemiol **46**(5): 1410-1420. | unrelated exposure |
| 1. Jacobs, S., et al. (2017). "Dietary Patterns Derived by Reduced Rank Regression Are Inversely Associated with Type 2 Diabetes Risk across 5 Ethnic Groups in the Multiethnic Cohort." Curr Dev Nutr **1**(5): e000620. | unrelated exposure |
| 1. Hruby, A., et al. (2017). "Magnesium Intake, Quality of Carbohydrates, and Risk of Type 2 Diabetes: Results From Three U.S. Cohorts." Diabetes Care **40**(12): 1695-1702. | unrelated exposure |
| 1. Golozar, A., et al. (2017). "White rice intake and incidence of type-2 diabetes: analysis of two prospective cohort studies from Iran." BMC Public Health **17**(1): 1-11. | unrelated exposure |
| 1. Feldman, A. L., et al. (2017). "Change in lifestyle behaviors and diabetes risk: evidence from a population-based cohort study with 10 year follow-up." Int J Behav Nutr Phys Act **14**(1): 39. | unrelated exposure |
| 1. Wallin, A., et al. (2016). "Egg consumption and risk of type 2 diabetes: a prospective study and dose–response meta-analysis." Diabetologia **59**(6): 1204-1213. | unrelated exposure |
| 1. Satija, A., et al. (2016). "Plant-Based Dietary Patterns and Incidence of Type 2 Diabetes in US Men and Women: Results from Three Prospective Cohort Studies." PLoS Med **13**(6): e1002039. | unrelated exposure |
| 1. Pastorino, S., et al. (2016). "A high-fat, high-glycaemic index, low-fibre dietary pattern is prospectively associated with type 2 diabetes in a British birth cohort." Br J Nutr **115**(9): 1632-1642. | unrelated exposure |
| 1. Biskup, I., et al. (2016). "Plasma alkylresorcinols, biomarkers of whole-grain wheat and rye intake, and risk of type 2 diabetes in Scandinavian men and women." Am J Clin Nutr **104**(1): 88-96. | unrelated exposure |
| 1. Sar, S. and G. C. Marks (2015). "Estimated effects of white rice consumption and rice variety selection on incidence of type 2 diabetes in Cambodia." Public Health Nutr **18**(14): 2592-2599. | unrelated exposure |
| 1. Lacoppidan, S. A., et al. (2015). "Adherence to a healthy Nordic food index is associated with a lower risk of type-2 diabetes—the Danish diet, cancer and health cohort study." Nutrients **7**(10): 8633-8644. | unrelated exposure |
| 1. Kuijsten, A., et al. (2015). "Dietary fibre and incidence of type 2 diabetes in eight European countries: the EPIC-InterAct Study and a meta-analysis of prospective studies." Diabetologia **58**(7): 1394-1408. | unrelated exposure |
| 1. Ericson, U., et al. (2015). "Food sources of fat may clarify the inconsistent role of dietary fat intake for incidence of type 2 diabetes." American Journal of Clinical Nutrition **101**(5): 1065-1080. | unrelated exposure |
| 1. Dominguez, L. J., et al. (2015). "Association of a Dietary Score with Incident Type 2 Diabetes: The Dietary-Based Diabetes-Risk Score (DDS)." PLoS One **10**(11): e0141760. | unrelated exposure |
| 1. AlEssa, H. B., et al. (2015). "Carbohydrate quality and quantity and risk of type 2 diabetes in US women." Am J Clin Nutr **102**(6): 1543-1553. | unrelated exposure |
| 1. Vashum, K. P., et al. (2013). "Is dietary zinc protective for type 2 diabetes? Results from the Australian longitudinal study on women's health." BMC Endocrine Disorders **13**. | unrelated exposure |
| 1. Ericson, U., et al. (2013). "High intakes of protein and processed meat associate with increased incidence of type 2 diabetes." Br J Nutr **109**(6): 1143-1153. | unrelated exposure |
| 1. Mekary, R. A., et al. (2013). "Eating patterns and type 2 diabetes risk in older women: breakfast consumption and eating frequency." Am J Clin Nutr **98**(2): 436-443. | unrelated exposure |
| 1. Rossi, M., et al. (2013). "Mediterranean diet and glycaemic load in relation to incidence of type 2 diabetes: Results from the Greek cohort of the population-based European Prospective Investigation into Cancer and Nutrition (EPIC)." Diabetologia **56**(11): 2405-2413. | unrelated exposure |
| 1. Ford, E. S., et al. (2009). "Healthy living is the best revenge: findings from the European Prospective Investigation Into Cancer and Nutrition-Potsdam study." Arch Intern Med **169**(15): 1355-1362. | unrelated exposure |
| 1. Rayner, J., et al. (2020). "Carbohydrate restriction in midlife is associated with higher risk of type 2 diabetes among Australian women: A cohort study." Nutr Metab Cardiovasc Dis **30**(3): 400-409. | unrelated exposure |
| 1. Partula, V., et al. (2020). "Associations between consumption of dietary fibers and the risk of cardiovascular diseases, cancers, type 2 diabetes, and mortality in the prospective NutriNet-Santé cohort." Am J Clin Nutr **112**(1): 195-207. | unrelated exposure |
| 1. Xu, X., et al. (2019). "Can regular long-term breakfast cereals consumption benefits lower cardiovascular diseases and diabetes risk? A longitudinal population-based study." Ann Epidemiol **37**: 43-50.e43. | unrelated exposure |
| 1. Farhadnejad, H., et al. (2018). "The Association of Potato Intake With Risk for Incident Type 2 Diabetes in Adults." Can J Diabetes **42**(6): 613-618. | unrelated exposure |
| 1. Díaz-López, A., et al. (2016). "Dairy product consumption and risk of type 2 diabetes in an elderly Spanish Mediterranean population at high cardiovascular risk." Eur J Nutr **55**(1): 349-360. | unrelated exposure |
| 1. Anjana, R. M., et al. (2015). "Diabetes in Asian Indians-How much is preventable? Ten-year follow-up of the Chennai Urban Rural Epidemiology Study (CURES-142)." Diabetes Res Clin Pract **109**(2): 253-261. | unrelated exposure |
| 1. Domínguez, L. J., et al. (2013). "Similar prediction of total mortality, diabetes incidence and cardiovascular events using relative- and absolute-component Mediterranean diet score: the SUN cohort." Nutr Metab Cardiovasc Dis **23**(5): 451-458. | unrelated exposure |
| 1. Colditz, G. A., et al. (1992). "Diet and risk of clinical diabetes in women." Am J Clin Nutr **55**(5): 1018-1023. | unrelated exposure |
| 1. Weng, L. C., et al. (2012). "Lower intake of magnesium and dietary fiber increases the incidence of type 2 diabetes in Taiwanese." J Formos Med Assoc **111**(11): 651-659. | unrelated exposure |
| 1. Salmerón, J., et al. (1997). "Dietary fiber, glycemic load, and risk of non-insulin-dependent diabetes mellitus in women." Jama **277**(6): 472-477. | unrelated exposure |
| 1. André, P., et al. (2020). "The role of overweight in the association between the Mediterranean diet and the risk of type 2 diabetes mellitus: a mediation analysis among 21 585 UK biobank participants." Int J Epidemiol. | unrelated exposure |
| 1. Lee, K. W., et al. (2019). "Dietary carbohydrate quality and quantity in relation to the incidence of type 2 diabetes: A prospective cohort study of middle-aged and older Korean adults." Nutrition **57**: 245-251. | unrelated exposure |
| 1. Moslehi, N., et al. (2016). "Patterns of food consumption and risk of type 2 diabetes in an Iranian population: A nested case-control study." Nutrition and Dietetics **73**(2): 169-176. | unrelated exposure |
| 1. Koloverou, E., et al. (2016). "Adherence to Mediterranean diet and 10-year incidence (2002-2012) of diabetes: correlations with inflammatory and oxidative stress biomarkers in the ATTICA cohort study." Diabetes Metab Res Rev **32**(1): 73-81. | unrelated exposure |
| 1. van Dam, R. M., et al. (2002). "Dietary patterns and risk for type 2 diabetes mellitus in U.S. men." Ann Intern Med **136**(3): 201-209. | unrelated exposure |
| 1. Jiang, R., et al. (2002). "Nut and peanut butter consumption and risk of type 2 diabetes in women." Jama **288**(20): 2554-2560. | unrelated exposure |
| 1. Halton, T. L., et al. (2006). "Potato and french fry consumption and risk of type 2 diabetes in women." Am J Clin Nutr **83**(2): 284-290. | unrelated exposure |
| 1. The association of dietary macronutrients composition with the incidence of type 2 diabetes, using iso-energetic substitution models: Tehran Lipid and Glucose Study | unrelated to exposure |
| 1. McNaughton, S. A., et al. (2008). "Dietary patterns, insulin resistance, and incidence of type 2 diabetes in the Whitehall II Study." Diabetes Care **31**(7): 1343-1348. | unrelated exposure |
| 1. Bhupathiraju, S. N., et al. (2014). "Glycemic index, glycemic load, and risk of type 2 diabetes: results from 3 large US cohorts and an updated meta-analysis." Am J Clin Nutr **100**(1): 218-232. | unrelated exposure |
| 1. Baghdasarian, S., et al. (2018). "Dietary cholesterol intake is not associated with risk of type 2 diabetes in the framingham offspring study." Nutrients **10**(6). | unrelated exposure |
| 1. Muraki, I., et al. (2016). "Potato Consumption and Risk of Type 2 Diabetes: Results From Three Prospective Cohort Studies." Diabetes Care **39**(3): 376-384. | unrelated exposure |
| 1. Malik, V. S., et al. (2016). "Dietary Protein Intake and Risk of Type 2 Diabetes in US Men and Women." Am J Epidemiol **183**(8): 715-728. | unrelated exposure |
| 1. Influence of dietary patterns on type 2 diabetes mellitus in local residents aged 40 years and above in Songjiang district, Shangha. | unrelated to exposure |
| 1. Nettleton, J. A., et al. (2008). "Dietary patterns and risk of incident type 2 diabetes in the Multi-Ethnic Study of Atherosclerosis (MESA)." Diabetes Care **31**(9): 1777-1782. | unrelated exposure |
| 1. The relationship between gut microbiota, short-chain fatty acids and type 2 diabetes mellitus: the possible role of dietary fibre | unrelated to exposure |
| 1. Kochar, J., et al. (2007). "Breakfast cereals and risk of type 2 diabetes in the Physicians' Health Study I." Obesity (Silver Spring) **15**(12): 3039-3044. | unrelated exposure |
| 1. The role of overweight in the association between the Mediterranean diet and the risk of type 2 diabetes mellitus: a mediation analysis among 21 585 UK biobank participants | unrelated to exposure |
| 1. Do Cultural and Psychosocial Factors Contribute to Type 2 Diabetes Risk? A Look Into Vancouver's South Asian Community | unrelated to exposure |
| 1. Habitual intake of dietary L-arginine in relation to risk of type 2 diabetes: a prospective study | unrelated to exposure |
| 1. A review of recent findings on meal sequence: An attractive dietary approach to prevention and management of type 2 diabetes | unrelated to exposure |
| 1. Replacement of red and processed meat with other food sources of protein and the risk of type 2 diabetes in European populations: The epic-interact study | unrelated to exposure |
| 1. Consumption of Beverages Containing Low-Calorie Sweeteners, Diet, and Cardiometabolic Health in Youth With Type 2 Diabetes | unrelated to exposure |
| 1. Maternal High-Fiber Diet Protects Offspring against Type 2 Diabetes | unrelated to exposure |
| 1. Manganese intake from foods and beverages is associated with a reduced risk of type 2 diabetes | unrelated to exposure |
| 1. Consumption of dairy products in relation to type 2 diabetes mellitus in chinese people: The henan rural cohort study and an updated meta-analysis | unrelated to exposure |
| 1. Diet and exercise in the prevention and treatment of type 2 diabetes mellitus | unrelated to exposure |
| 1. Association of dietary fiber intake with newly-diagnosed type 2 diabetes mellitus in middle-aged Chinese population | unrelated to exposure |
| 1. Replacing Red Meat with Other Nonmeat Food Sources of Protein is Associated with a Reduced Risk of Type 2 Diabetes in a Danish Cohort of Middle-Aged Adults | unrelated to exposure |
| 1. Association of magnesium intake with type 2 diabetes and total stroke: an updated systematic review and meta-analysis | unrelated to exposure |
| 1. A systematic review and dose-response meta-analysis of exposure to environmental selenium and the risk of type 2 diabetes in nonexperimental studies | unrelated to exposure |
| 1. Incidence of end-stage renal disease and risk factors for progression of renal dysfunction in Japanese patients with type 2 diabetes: the Fukuoka Diabetes Registry | unrelated to exposure and outcome |
| 1. Incidence of stroke and its association with glycemic control and lifestyle in Japanese patients with type 2 diabetes mellitus: The Fukuoka diabetes registry | unrelated to exposure and outcome |
| 1. Prospective study of a diabetes risk reduction diet and the risk of breast cancer | unrelated to exposure and outcome |
| 1. The effects of 6 mo of supplementation with probiotics and synbiotics on gut microbiota in the adults with prediabetes: A double blind randomized clinical trial | unrelated to exposure and outcome |
| 1. Facilitators and barriers to preparing and offering whole grains to children diagnosed with prediabetes: qualitative interviews with low-income caregivers | unrelated to exposure and outcome |
| 1. Dietary Fibre Intake in Type 2 and New-Onset Prediabetes/Diabetes after Acute Pancreatitis: A Nested Cross-Sectional Study | unrelated to exposure and outcome |
| 1. Sociodemographic Factors Associated with Adherence to Dietary Guidelines in Women with Gestational Diabetes: A Cohort Study | unrelated to exposure and outcome |
| 1. Relevance of dietary glycemic index, glycemic load and fiber intake before and during pregnancy for the risk of gestational diabetes mellitus and maternal glucose homeostasis | unrelated to exposure and outcome |
| 1. Effect of Eating Glutinous Brown Rice Twice a Day for 6 Weeks on Serum 1,5-Anhydroglucitol in Japanese Subjects without Diabetes | unrelated to exposure and outcome |
| 1. Dietary intake of carbohydrates in pregnant women with type 1 diabetes-A narrative review | unrelated to exposure and outcome |
| 1. Frequency of consumption of balanced meals, bodyweight gain and incident risk of glucose intolerance in Japanese men and women: A cohort study | unrelated to exposure and outcome |
| 1. Effects of nutrition education using a food-based approach, carbohydrate counting or routine care in type 1 diabetes: 12 months prospective randomized trial | unrelated to exposure and outcome |
| 1. Relationship between dietary carbohydrate quality index and metabolic syndrome among type 2 diabetes mellitus subjects: a case-control study from Ghana | unrelated to exposure and outcome |
| 1. Dietary metabolite profiling brings new insight into the relationship between nutrition and metabolic risk: An IMI DIRECT study | unrelated to exposure and outcome |
| 1. Dietary patterns and risk of gestational diabetes mellitus: A systematic review and meta-analysis of cohort studies | unrelated to exposure and outcome |
| 1. Dietary fibre for the prevention of cardiovascular disease | unrelated to exposure and outcome |
| 1. Effects of a diabetes education intervention on diabetes-related factors among Mexican-origin Hispanics | unrelated to exposure and outcome |
| 1. South Indian cuisine with low glycemic index ingredients reduces cardiovascular risk factors in subjects with type 2 diabetes | unrelated to exposure and outcome |
| 1. Red and processed meats and health risks: How strong is the evidence? | unrelated to exposure and outcome |
| 1. Foods contributing to nutrients intake and assessment of nutritional status in pre-dialysis patients: A cross-sectional study | unrelated to exposure and outcome |
| 1. Feel4Diabetes healthy diet score: Development and evaluation of clinical validity | unrelated to exposure and outcome |
| 1. A prospective study on total protein, plant protein and animal protein in relation to the risk of incident chronic kidney disease | unrelated to exposure and outcome |
| 1. The association between dietary glycemic index and load and risk of gestational diabetes mellitus: A prospective study | unrelated to exposure and outcome |
| 1. Healthcare workers with diabetes need for more attention in COVID-19 Outbreak | unrelated to exposure and outcome |
| 1. Vegetable diversity in relation with subclinical atherosclerosis and 15-year atherosclerotic vascular disease deaths in older adult women | unrelated to exposure and outcome |
| 1. Antidiabetic and hypolipidemic action of finger millet (Eleusine coracana) enriched probiotic fermented milk: An in vivo rat study | unrelated to exposure and outcome |
| 1. The association between dietary and skin advanced glycation end products: The Rotterdam Study | unrelated to exposure and outcome |
| 1. Associations of specific dietary protein with longitudinal insulin resistance, prediabetes and type 2 diabetes: The Rotterdam Study | unrelated to exposure and outcome |
| 1. Dietary total antioxidant capacity and its association with sleep, stress, anxiety, and depression score: A cross-sectional study among diabetic women | unrelated to exposure and outcome |
| 1. Total and added sugar intakes, sugar types, and cancer risk: Results from the prospective NutriNet-Santé cohort | unrelated to exposure and outcome |
| 1. Association between dietary glycemic load during first trimester and the risk of gestational diabetes mellitus: a prospective study | unrelated to exposure and outcome |
| 1. Self-reported eating speed and incidence of gestational diabetes mellitus: The Japan environment and children’s study | unrelated to exposure and outcome |
| 1. Physicochemical properties and health benefits of pistachio nuts: A comprehensive review | unrelated to exposure and outcome |
| 1. Carbohydrate quality, glycemic index, glycemic load and cardiometabolic risks in the US, Europe and Asia: A dose–response meta-analysis | unrelated to exposure and outcome |
| 1. The quality of dietary carbohydrate and fat is associated with better metabolic control in persons with type 1 and type 2 diabetes | unrelated to exposure and outcome |
| 1. Adherence to dietary approaches to stop hypertension (DASH) and Mediterranean dietary patterns in relation to cardiovascular risk factors in older adults | unrelated to exposure and outcome |
| 1. Diet and Lifestyle as Risk Factors for Carotid Artery Disease: A Prospective Cohort Study | unrelated to exposure and outcome |
| 1. Awareness on effect of high fat diet among adolescents | unrelated to exposure and outcome |
| 1. Diabetes mellitus and dietary starch in perspective of blood glycaemic control | unrelated to exposure and outcome |
| 1. Dietary patterns and dietary adaptations in women with and without gestational diabetes: Evidence from the growing up in New Zealand study | unrelated to exposure and outcome |
| 1. Influence of dietary pattern on human immunity | unrelated to exposure and outcome |
| 1. Combined associations of body mass index and adherence to a Mediterranean-like diet with all-cause and cardiovascular mortality: A cohort study | unrelated to exposure and outcome |
| 1. Assessing the association between optimal energy intake and all-cause mortality in older patients with diabetes mellitus using the Japanese Elderly Diabetes Intervention Trial | unrelated to exposure and outcome |
| 1. Intermittent fasting 5:2 diet: What is the macronutrient and micronutrient intake and composition? | unrelated to exposure and outcome |
| 1. Association between dietary patterns and prediabetes risk in a middle-Aged Chinese population | unrelated to exposure and outcome |
| 1. Spinach and quinoa -prospective food sources of biologically active substances | unrelated to exposure and outcome |
| 1. Eating fast is associated with nonalcoholic fatty liver disease in men but not in women with type 2 diabetes: A cross-sectional study | unrelated to exposure and outcome |
| 1. Nutritional and metabolic regulation of the metabolite dimethylguanidino valeric acid: an early marker of cardiometabolic disease | unrelated to exposure and outcome |
| 1. Eating and healthy ageing: a longitudinal study on the association between food consumption, memory loss and its comorbidities | unrelated to exposure and outcome |
| 1. Does daily dietary intake affect diabetic retinopathy progression? 10-year results from the 45 and up Study | unrelated to exposure and outcome |
| 1. Associations of choline-related nutrients with cardiometabolic and all-cause mortality: Results from 3 prospective cohort studies of blacks, whites, and Chinese | unrelated to exposure and outcome |
| 1. Carbohydrates: Separating fact from fiction | unrelated to exposure and outcome |
| 1. Dietary phenotype and advanced glycation end-products predict WTC-obstructive airways disease: a longitudinal observational study | unrelated to exposure and outcome |
| 1. Stroke and diets-A review | unrelated to exposure and outcome |
| 1. Diet and Prognosis in Women with Breast Cancer | unrelated to exposure and outcome |
| 1. Dietary recommendations of magnesium for cardiovascular prevention and treatment. A position paper of the israel heart society and the israel dietetic association | unrelated to exposure and outcome |
| 1. Gut Microbiota and Type 1 Diabetes Mellitus: The Effect of Mediterranean Diet | unrelated to exposure and outcome |
| 1. Diabetes Risk Reduction Diet and Endometrial Cancer Risk | unrelated to exposure and outcome |
| 1. Effects of prebiotic consumption on serum intestinal fatty acid-binding protein levels in patients with diabetes: A case-control study | unrelated to exposure and outcome |
| 1. Associations of dietary patterns and nutrients with glycated hemoglobin in participants with and without type 1 diabetes | unrelated to exposure and outcome |
| 1. Associations of dietary patterns and nutrients with coronary artery calcification and pericardial adiposity in a longitudinal study of adults with and without type 1 diabetes | unrelated to exposure and outcome |
| 1. Effect of a High-Protein High-Fibre Nutritional Supplement on Lipid Profile in Overweight/Obese Adults with Type 2 Diabetes Mellitus: A 24-Week Randomized Controlled Trial | unrelated to exposure and outcome |
| 1. Constipation and glycemic control | unrelated to exposure and outcome |
| 1. Association of maternal pre-pregnancy dietary intake with adverse maternal and neonatal outcomes: A systematic review and meta-analysis of prospective studies | unrelated to exposure and outcome |
| 1. Scoping review of Paleolithic dietary patterns: A definition proposal | unrelated to exposure and outcome |
| 1. Evaluating an Intervention to Increase Cereal Fiber Intake in Children: A Randomized Controlled Feasibility Trial | unrelated to exposure and outcome |
| 1. Effects of whey protein and dietary fiber intake on insulin sensitivity, body composition, energy expenditure, blood pressure, and appetite in subjects with abdominal obesity | unrelated to exposure and outcome |
| 1. Higher oxidative balance score is associated with better glycemic control among Iranian adults with type-2 diabetes | unrelated to exposure and outcome |
| 1. Maternal food consumption during late pregnancy and offspring risk of islet autoimmunity and type 1 diabetes | unrelated to exposure and outcome |
| 1. A plant-based meal affects thalamus perfusion differently than an energy- and macronutrient-matched conventional meal in men with type 2 diabetes, overweight/obese, and healthy men: A three-group randomized crossover study | unrelated to exposure and outcome |
| 1. The modulation of gut microbiota composition in the pathophysiology of gestational diabetes mellitus: A systematic review | unrelated to exposure and outcome |
| 1. Pre-gestational consumption of ultra-processed foods and risk of gestational diabetes in a mediterranean cohort. The SUN project | unrelated to exposure and outcome |
| 1. Mid-pregnancy consumption of fruit, vegetable and fruit juice and the risk of gestational diabetes mellitus: A correlation study | unrelated to exposure and outcome |
| 1. Using a paleo ratio to assess adherence to paleolithic dietary recommendations in a randomized controlled trial of individuals with type 2 diabetes | unrelated to exposure and outcome |
| 1. The role of dietary diversity in preventing metabolic-related outcomes: Findings from a systematic review | unrelated to exposure and outcome |
| 1. Low circulating arachidonic acid is associated with macroalbuminuria in diabetic patients: a cross-sectional examination of the KAMOGAWA-DM cohort study | unrelated to exposure and outcome |
| 1. Mediterranean, dash, and alternate healthy eating index dietary patterns and risk of death in the physicians’ health study | unrelated to exposure and outcome |
| 1. Low Mediterranean Diet scores are associated with reduced kidney function and health related quality of life but not other markers of cardiovascular risk in adults with diabetes and chronic kidney disease | unrelated to exposure and outcome |
| 1. Association between Fruit and Vegetable Intakes and Mental Health in the Australian Diabetes Obesity and Lifestyle Cohort | unrelated to exposure and outcome |
| 1. Nutrient patterns and depressive symptoms among Australian adults | unrelated to exposure and outcome |
| 1. Walnut consumption and cardiac phenotypes: The Coronary Artery Risk Development in Young Adults (CARDIA) study | unrelated to exposure and outcome |
| 1. The association of the Mediterranean diet with heart failure risk in a Dutch population | unrelated to exposure and outcome |
| 1. Habitual miso (Fermented soybean paste) consumption is associated with a low prevalence of sarcopenia in patients with type 2 diabetes: A cross-sectional study | unrelated to exposure and outcome |
| 1. The Role of High-Content Complex Dietary Fiber in Medical Nutrition Therapy for Gestational Diabetes Mellitus | unrelated to exposure and outcome |
| 1. Vaccaro, J. A. and F. G. Huffman (2011). "Monounsaturated fatty acid, carbohydrate intake, and diabetes status are associated with arterial pulse pressure." Nutr J **10**: 126. | unrelated to exposure and outcome |
| 1. Yamakawa, T., et al. (2019). "Dietary survey in Japanese patients with type 2 diabetes and the influence of dietary carbohydrate on glycated hemoglobin: The Sleep and Food Registry in Kanagawa study." J Diabetes Investig **10**(2): 309-317. | unrelated to exposure and outcome |
| 1. Dong, Y., et al. (2019). "Total, insoluble, and soluble dietary fiber intake and insulin resistance and blood pressure in adolescents." Eur J Clin Nutr **73**(8): 1172-1178. | unrelated to exposure and outcome |
| 1. den Biggelaar, L., et al. (2019). "Prospective associations of dietary carbohydrate, fat, and protein intake with β-cell function in the CODAM study." Eur J Nutr **58**(2): 597-608. | unrelated to exposure and outcome |
| 1. Morimoto, N., et al. (2018). "Association between dietary fibre:carbohydrate intake ratio and insulin resistance in Japanese adults without type 2 diabetes." Br J Nutr **119**(6): 620-628. | unrelated to exposure and outcome |
| 1. Jin, Y., et al. (2018). "Vegetarian Diets Are Associated with Selected Cardiometabolic Risk Factors among Middle-Older Aged South Asians in the United States." J Nutr **148**(12): 1954-1960. | unrelated to exposure and outcome |
| 1. Voortman, T., et al. (2017). "Adherence to the 2015 Dutch dietary guidelines and risk of non-communicable diseases and mortality in the Rotterdam Study." Eur J Epidemiol **32**(11): 993-1005. | unrelated to exposure and outcome |
| 1. Horikawa, C., et al. (2017). "Is the proportion of carbohydrate intake associated with the incidence of diabetes complications?—an analysis of the Japan diabetes complications study." Nutrients **9**(2). | unrelated to exposure and outcome |
| 1. López-Ortiz, M. M., et al. (2016). "Analysis of the interaction between transcription factor 7-like 2 genetic variants with nopal and wholegrain fibre intake: effects on anthropometric and metabolic characteristics in type 2 diabetes patients." Br J Nutr **116**(6): 969-978. | unrelated to exposure and outcome |
| 1. Heraclides, A., et al. (2016). "Investigation of gene–diet interactions in the incretin system and risk of type 2 diabetes: the EPIC-InterAct study." Diabetologia **59**(12): 2613-2621. | unrelated to exposure and outcome |
| 1. Conklin, A. I., et al. (2016). "Dietary Diversity, Diet Cost, and Incidence of Type 2 Diabetes in the United Kingdom: A Prospective Cohort Study." PLoS Med **13**(7). | unrelated to exposure and outcome |
| 1. Sjaarda, L. A., et al. (2015). "Dietary carbohydrate intake does not impact insulin resistance or androgens in healthy, eumenorrheic women." Journal of Clinical Endocrinology and Metabolism **100**(8): 2979-2986. | unrelated to exposure and outcome |
| 1. White, J., et al. (2014). "Dietary risk factors for the development of insulin resistance in adolescent girls: a 3-year prospective study." Public Health Nutr **17**(2): 361-368. | unrelated to exposure and outcome |
| 1. Kyrø, C., et al. (2014). "Plasma alkylresorcinol concentrations, biomarkers of whole-grain wheat and rye intake, in the European Prospective Investigation into Cancer and Nutrition (EPIC) cohort." Br J Nutr **111**(10): 1881-1890. | unrelated to exposure and outcome |
| 1. Hruby, A., et al. (2014). "Higher magnesium intake reduces risk of impaired glucose and insulin metabolism and progression from prediabetes to diabetes in middle-aged americans." Diabetes Care **37**(2): 419-427. | unrelated to exposure and outcome |
| 1. Van Rompay, M. I., et al. (2013). "Carbohydrate nutrition differs by diabetes status and is associated with dyslipidemia in Boston Puerto Rican adults without diabetes." J Nutr **143**(2): 182-188. | unrelated to exposure and outcome |
| 1. Magnusdottir, O. K., et al. (2013). "Plasma alkylresorcinols reflect important whole-grain components of a healthy Nordic diet." J Nutr **143**(9): 1383-1390. | unrelated to exposure and outcome |
| 1. Mayer-Davis, E. J., et al. (2006). "Dietary Intake among Youth with Diabetes: The SEARCH for Diabetes in Youth Study." J Am Diet Assoc **106**(5): 689-697. | unrelated to exposure and outcome |
| 1. Tajima, R., et al. (2017). "Carbohydrate intake during early pregnancy is inversely associated with abnormal glucose challenge test results in Japanese pregnant women." Diabetes Metab Res Rev **33**(6). | unrelated to exposure and outcome |
| 1. Różańska, D., et al. (2016). "Assessment of glycemic load and intake of carbohydrates in the diet of Wroclaw Medical University students (Poland)." Rocz Panstw Zakl Hig **67**(3): 301-308. | unrelated to exposure and outcome |
| 1. Jenkins, D. J., et al. (2002). "High-complex carbohydrate or lente carbohydrate foods?" Am J Med **113 Suppl 9B**: 30s-37s. | unrelated to exposure and outcome |
| 1. Tuomilehto, J., et al. (2001). "Lifestyle changes can prevent the development of diabetes mellitus." Cmaj **164**(13): 1885. | unrelated to exposure and outcome |
| 1. Evans, M. F. (2002). "Can we prevent high-risk patients from getting type 2 diabetes?" Canadian Family Physician **48**(FEB.): 279-281. | unrelated to exposure and outcome |
| 1. Gaesser, G. A. (2019). "Perspective: Refined Grains and Health: Genuine Risk, or Guilt by Association?" Adv Nutr **10**(3): 361-371. | unrelated to exposure and outcome |
| 1. Dietary fiber intake, dietary glycemic load, and the risk of gestational diabetes mellitus during the second trimester: A nested case-control study | unrelated to exposure and outcome |
| 1. Diabetes Risk Reduction Diet and Survival after Breast Cancer Diagnosis | unrelated to exposure and outcome |
| 1. Remote Peer Learning Between US and Cambodian Lay Health Workers to Improve Outcomes for Cambodians with Type 2 Diabetes: a Pilot Study | unrelated to exposure and outcome |
| 1. Associations of Dietary Patterns and Nutrients with Glycated Hemoglobin in Participants with and without Type 1 Diabetes | unrelated to exposure and outcome |
| 1. McKeown, N. M., et al. (2002). "Whole-grain intake is favorably associated with metabolic risk factors for type 2 diabetes and cardiovascular disease in the Framingham Offspring Study." Am J Clin Nutr **76**(2): 390-398. | cross-sectional study |
| 1. Lutsey, P. L., et al. (2007). "Whole grain intake and its cross-sectional association with obesity, insulin resistance, inflammation, diabetes and subclinical CVD: The MESA Study." Br J Nutr **98**(2): 397-405. | cross-sectional study |
| 1. Murtaugh, M. A., et al. (2003). "Epidemiological support for the protection of whole grains against diabetes." Proceedings of the Nutrition Society **62**(1): 143-149. | reviews and meta-analyses |
| 1. Oat Intake and Risk of Type 2 Diabetes, Cardiovascular Disease and All-Cause Mortality: A Systematic Review and Meta-Analysis | reviews and meta-analyses |
| 1. Preventive Role of Diet Interventions and Dietary Factors in Type 2 Diabetes Mellitus: An Umbrella Review | reviews and meta-analyses |
| 1. Liu, S. (2003). "Whole-grain foods, dietary fiber, and type 2 diabetes: searching for a kernel of truth." Am J Clin Nutr **77**(3): 527-529. | reviews and meta-analyses |
| 1. Jones, J. M., et al. (2020). "Perspective: Whole and Refined Grains and Health-Evidence Supporting "Make Half Your Grains Whole"." Adv Nutr **11**(3): 492-506. | reviews and meta-analyses |
| 1. Whole grain consumption and human health: an umbrella review of observational studies | reviews and meta-analyses |
| 1. Frølich, W., et al. (2013). "Whole grain foods and health - A Scandinavian perspective." Food and Nutrition Research **57**. | reviews and meta-analyses |
| 1. Bach Knudsen, K. E., et al. (2016). Mechanisms Whereby Whole Grain Cereals Modulate the Prevention of Type 2 Diabetes. Molecular Nutrition and Diabetes: A Volume in the Molecular Nutrition Series**:** 87-103. | reviews and meta-analyses |
| 1. Wolever, T. M. S. (2013). "Do whole grain cereals really reduce LDL cholesterol by 0.72 mmol/L?" Journal of Nutrition **143**(9): 1521-1522. | Letter |
| 1. Liu, S., et al. (2000). "A prospective study of whole-grain intake and risk of type 2 diabetes mellitus in US women." Am J Public Health **90**(9): 1409-1415. | same population |
| 1. Fung, T. T., et al. (2002). "Whole-grain intake and the risk of type 2 diabetes: a prospective study in men." Am J Clin Nutr **76**(3): 535-540. | same population |
| 1. Schulze, M. B., et al. (2007). "An accurate risk score based on anthropometric, dietary, and lifestyle factors to predict the development of type 2 diabetes." Diabetes Care **30**(3): 510-515. | same population |
| 1. Sun, Q., et al. (2010). "White rice, brown rice, and risk of type 2 diabetes in US men and women." Arch Intern Med **170**(11): 961-969. | same population |
| 1. von Ruesten, A., et al. (2013). "Diet and risk of chronic diseases: results from the first 8 years of follow-up in the EPIC-Potsdam study." Eur J Clin Nutr **67**(4): 412-419. | same population |

**Supplemental Table 3:** Risk of bias assessment using the modified Newcastle-Ottawa scale of included cohort studies in systematic review of whole grain intake and risk of type 2 diabetes.

|  | Total score | |  |  |  |  |  |  |  |  |  |
| --- | --- | --- | --- | --- | --- | --- | --- | --- | --- | --- | --- |
|  | Representativeness of the exposed cohort | Selection of the non-exposed cohort | | Ascertainment of exposure | Demonstration that outcome of interest was not present at start of study | Study controls for energy intake | Study controls for any additional factor | Assessment of outcome | Was follow-up long enough for outcomes to occur (>10 years) | Adequacy of follow up of cohorts (loss-to-follow up <20%) | **Total score** |
| Meyer et al. (1) | * | * | | * | * | * | * | - | - | * | 7 |
| Montonen et al. (2) | * | * | | * | * | * | * | * | * | - | 8 |
| Van Dam et al. (3) | - | * | | * | * | * | * | - | - | * | 7 |
| Fisher et al. (4) | * | - | | * | - | * | * | * | - | - | 5 |
| Wirstrom et al. (5) | * | * | | * | * | - | * | * | - | - | 6 |
| Parker et al. (6) | * | * | | * | * | * | * | - | - | - | 6 |
| Ericson et al. (7) | * | * | | * | * | * | * | * | * | * | 8 |
| Kyro et al. (8) | * | * | | * | * | - | * | * | * | - | 7 |
| Hu et al. (9) | - | * | | * | * | - | * | - | * | - | 7 |
| Hu et al. (10) | - | * | | * | * | - | * | - | * | - | 7 |
| Hu et al. (11) | - | * | | * | * | - | * | - | * | - | 7 |

**Supplemental Table 4**: Leave-one-out sensitivity analysis of the association between whole grain intake and risk of type 2 diabetes.

| **Supplemental Table 5**: Relative risks of non-linear dose-response association between whole grain intake and risk of type 2 diabetes. | | |
| --- | --- | --- |
| Dose (gram/day) | RR | (95% CI) |
| 2.5 | 1.00 | (1.00-1.00) |
| 5 | 0.98 | (0.98-0.99) |
| 7.5 | 0.97 | (0.96-0.97) |
| 10 | 0.95 | (0.95-0.96) |
| 12.5 | 0.94 | (0.93-0.95) |
| 17.5 | 0.91 | (0.90-0.92) |
| 20 | 0.90 | (0.88-0.91) |
| 25 | 0.87 | (0.86-0.89) |
| 27.5 | 0.86 | (0.84-0.88) |
| 30 | 0.85 | (0.83-0.87) |
| 35 | 0.84 | (0.82-0.86) |
| 37.5 | 0.83 | (0.81-0.85) |
| 40 | 0.83 | (0.80-0.85) |
| 42.5 | 0.82 | (0.80-0.84) |
| 45 | 0.82 | (0.79-0.84) |
| 50 | 0.81 | (0.79-0.83) |
| 60 | 0.80 | (0.78-0.82) |
| 62.5 | 0.80 | (0.78-0.82) |
| 65 | 0.80 | (0.77-0.82) |
| 70 | 0.80 | (0.77-0.82) |
| 75 | 0.80 | (0.77-0.82) |
| 80 | 0.79 | (0.77-0.82) |
| 85 | 0.79 | (0.76-0.83) |
| 130 | 0.78 | (0.73-0.84) |
| 147.5 | 0.78 | (0.72-0.85) |

| **Supplemental Table 6**: Whole grain definition and amount of serving size in included studies. | | | | |
| --- | --- | --- | --- | --- |
| study name | Study population | WG definition | WG amount | WG unit |
| Meyer et al, 2000 | IWHS | WG foods reported by FFQ | - | servings per week |
| Montonen et al, 2003 | Finnish prospective study | whole-grain food group contained rye bread, rye crisp bread, and all whole-grain flours and other products (rye, whole wheat, wheat germ, rolled oats, barley, millet, and buckwheat) derived from different grain foods [eg, porridge, gruel, and Karelian pie (a national food of rice pudding baked in rye pastry and greased by butter), breads prepared from mixtures of whole grains and refined grains with proportion of whole-grain flours 25–50% | - | gram per day |
| Van Dam et al, 2006 | BWHS | dark breads, such as wheat, rye, pumpernickel in amount of 2 slices per serving | 2 slices per serving | serving per day |
| Fisher et al, 2009 | (EPIC)-Potsdam cohort | The sum of whole-grain bread, whole-grain bread rolls, and whole-grain cereals  one portion is considered as 50 g. | 1 serving:50 grams | gram per day |
| Wirstrom et al, 2012 | - | all food items that contained $50% of whole grain per serving (ie, crisp bread, whole-meal bread, oatmeal, and muesli) | - | gram per day  WG as ingredient |
| Parker et al, 2013 | WHIS | WG foods according to FFQ | - | serving per day |
| Ericson et al, 2018 | MDC | WG products | fiber-rich soft bread: 50gram/serving  fiber-rich crispbread: 30gram/serving  breakfast cereals:25gram/serving | serving per day |
| Kyro et al, 2018 | DDC | wheat, oats, rye, barley, rice, millet, corn/maize(dried), triticale, and sorghum/durra | 1serving of WG: 16 gram  1serving of WG products:  rye bread = 50 g (1 slice), whole-grain bread = 40 g (1 slice), and oatmeal/muesli = 50 g | gram per day  WG as ingredient |
| Hu et al, 2020 | NHS I | whole wheat and whole wheat flour, whole oats and whole oat flour, whole cornmeal and whole corn flour, whole rye and whole rye flour, whole barley, bulgur, buckwheat, brown rice and brown rice flour, popcorn, amaranth, and psyllium | 1serving: 16 gram WG as ingredient | serving per day  WG as ingredient |
| Hu et al, 2020 | NHS II | whole wheat and whole wheat flour, whole oats and whole oat flour, whole cornmeal and whole corn flour, whole rye and whole rye flour, whole barley, bulgur, buckwheat, brown rice and brown rice flour, popcorn, amaranth, and psyllium | 1serving: 16 gram WG as ingredient | serving per day  WG as ingredient |
| Hu et al, 2020 | HPFS | whole wheat and whole wheat flour, whole oats and whole oat flour, whole cornmeal and whole corn flour, whole rye and whole rye flour, whole barley, bulgur, buckwheat, brown rice and brown rice flour, popcorn, amaranth, and psyllium | 1serving: 16 gram WG as ingredient | serving per day  WG as ingredient |

**Supplemental Figures**

**Supplemental Figure 1.** Leave-one-out sensitivity analysis of the association between whole grain intake and risk of type 2 diabetes.


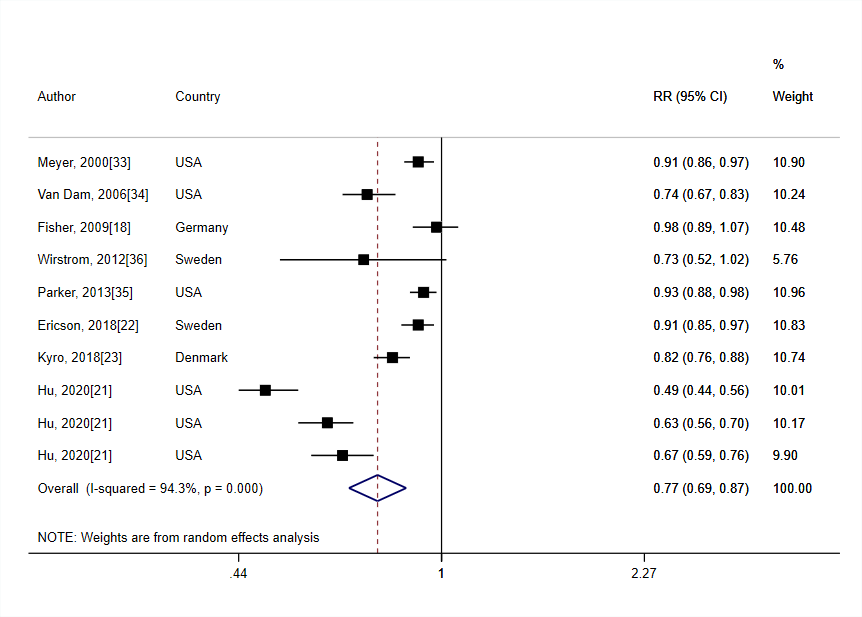


**Supplemental Figure 2.** Relative risk and 95% confidence intervals (CIs) of type 2 diabetes for 50 gram/day of whole grain intake.


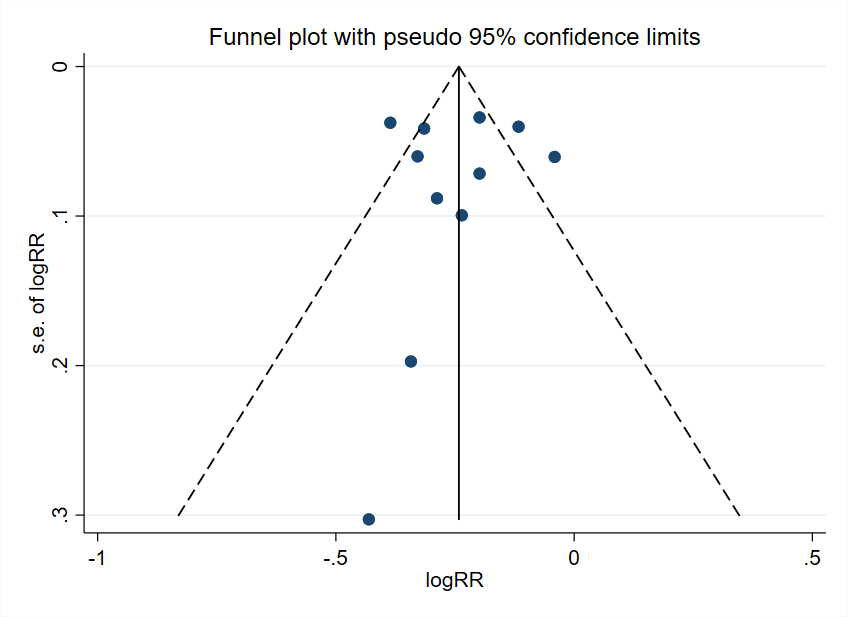


**Supplemental Figure 3.** Funnel plot of the meta-analysis of published studies. Each plotted point represents the standard error and standardized mean difference between high and low intakes of whole grain for a single study. The triangle represents the region where 95% of the data points would lie in the absence of a publication bias. The vertical line represents the average standardized mean difference of 0.30 found in the meta-analysis.
